# Tissue‐specific expression of insulin receptor isoforms in obesity/type 2 diabetes mouse models

**DOI:** 10.1111/jcmm.16452

**Published:** 2021-03-19

**Authors:** Noah Moruzzi, Francesca Lazzeri‐Barcelo, Ismael Valladolid‐Acebes, Tilo Moede, Meike Paschen, Barbara Leibiger, Per‐Olof Berggren, Ingo B. Leibiger

**Affiliations:** ^1^ Department of Molecular Medicine and Surgery The Rolf Luft Research Center for Diabetes and Endocrinology Karolinska Institutet Karolinska University Hospital Stockholm Sweden

**Keywords:** diabetes, insulin receptor, IR‐A, IR‐B, isoforms, obesity

## Abstract

The two insulin receptor (IR) isoforms IR‐A and IR‐B are responsible for the pleiotropic actions of insulin and insulin‐like growth factors. Consequently, changes in IR isoform expression and in the bioavailability of their ligands will impact on IR‐mediated functions. Although alteration of IR isoform expression has been linked to insulin resistance, knowledge of IR isoform expression and mechanisms underlying tissue/cell‐type‐specific changes in metabolic disease are lacking. Using mouse models of obesity/diabetes and measuring the mRNA of the IR isoforms and mRNA/protein levels of total IR, we provide a data set of IR isoform expression pattern that documents changes in a tissue‐dependent manner. Combining tissue fractionation and a new in situ mRNA hybridization technology to visualize the IR isoforms at cellular resolution, we explored the mechanism underlying the change in IR isoform expression in perigonadal adipose tissue, which is mainly caused by tissue remodelling, rather than by a shift in IR alternative splicing in a particular cell type, e.g. adipocytes.

## 1 INTRODUCTION

Insulin is essential for life as it regulates glucose homeostasis, directs metabolic substrate utilization and orchestrates a series of cellular actions such as cell growth, cell survival and activation of biosynthetic pathways.^1^, ^2^ The first node in insulin signalling is the insulin receptor (IR). Two IR isoforms, IR‐A and IR‐B, are generated by alternative splicing of exon 11 of the IR pre‐mRNA.^3^, ^4^, ^5^ IR‐A lacks and IR‐B contains the 12 amino acids encoded by exon 11, which are located in the C‐terminus of the extracellular insulin‐binding α‐chain of the receptor.

Current knowledge shows that IR‐A has high affinity for insulin, proinsulin and IGF2, while IR‐B is a more insulin‐specific receptor.^2^ Hence, the nature of the expressed IR isoform in a specific cell will determine the binding of different ligands, the subsequent signal transduction and thereby the biological effects. In the pancreatic β‐cell, the two IR isoforms co‐exist and are responsible for the activation of diverse signalling pathways.^6^, ^7^, ^8^, ^9^ While whole tissue studies showed that IR‐B is predominantly expressed in ‘classical’ insulin target tissues such as liver and fat, IR‐A seems to be ubiquitously expressed and is also associated with embryonic tissues and cancer cells.^2^ However, the knowledge of cell type‐specific IR isoform expression in different tissues/cells in health and disease is poor due to the lack of technical tools that selectively recognize IR‐A/IR‐B proteins or mRNAs at cellular resolution. Consequently, the expression pattern of IR isoforms in health and disease and their biological relevance remains elusive in most cell types. In white adipose tissue, a change in IR isoform gene expression during the development of obesity and/or type 2 diabetes (T2DM) has been hypothesized to cause or contribute to insulin resistance,^10^, ^11^ while in muscle the reported changes in IR isoforms mRNA were contradictory.^12^, ^13^, ^14^ Astonishingly, basic information of the tissue/cell‐type‐specific expression pattern of the two IR isoforms during metabolic disease is lacking even in the standard mouse model in biomedical research, the C57BL/6J mouse.

In this work, by measuring the amounts and ratio of IR isoform mRNAs as well as the mRNA and protein levels of total IR, we have generated a new informative dataset of IR expression in a broad spectrum of tissues in healthy C57BL/6J mice and genetic and dietary mouse models of obesity, insulin resistance and T2DM. By combining tissue fractionation with a novel mRNA in situ hybridization technology, we studied the mechanisms underlying the change in IR isoform expression in perigonadal adipose tissue (pGAT) as an example of tissues composed of multiple cell types.

## 2 MATERIALS AND METHODS

### 2.1 Mouse models and diets

Experiments were conducted on male mice allowed to adapt to the animal facility for at least one week prior to the start of diet intervention. Animals were group‐housed on a 12/12‐hour dark/light cycle with ad libitum access to food and water. Experimental groups are described in Supporting information. Animal experiments were performed in accordance to the Animal Experiment Ethics Committee at Karolinska Institutet.

### 2.2 mRNA processing from tissues

Animals were sacrificed by cervical dislocation and organs were quickly dissected. Tissues were submerged in RNAlater (ThermoFisher, Waltham, MA, USA) unless otherwise stated, incubated at 4°C for 24 hours and stored at −80°C. Brain tissues were snap‐frozen and immediately stored at −80°C. The hypothalamus was dissected out from 200 µm thick sections using micro punches and stored at −80°C. Pancreatic islets were prepared after ductal injection of collagenase P (F. Hoffmann‐La Roche, Basel, Switzerland) as previously described^15^ and processed with RLT buffer (QIAGEN, Venlo, Netherlands). Organs portion, used for mRNA extraction, was consistent: heart: right ventricle; liver: first lobe; kidney: horizontal cut in the lower or upper segment; intestine: duodenum. For mRNA extraction, RNase lipid tissue mini kit (adipose tissue, liver, hypothalamus) or RNase plus mini kit (both from QIAGEN) were used. Extracted RNA concentration and purity were measured using a nanophotometer (Implen GmbH, Munich, Germany).

### 2.3 Primer design and method validation

Efficiency and specificity of the designed primers for IR‐A, IR‐B and total IR validation are shown in Figure S1. Plasmids containing IR‐A and IR‐B amplicons were generated by amplifying a portion of IR cDNA from mouse using the primer pair F_ CCCCAGGCCATCCCGAA; R_ TGTGCTCCTCCTGACTTGT. The IR‐A and IR‐B PCR products were separated on a 4% agarose gel, purified by using Purelink PCR purification kit (ThermoFisher), and inserted into TOPO 2.1 vector (TOPO TA cloning kit, ThermoFisher).

### 2.4 Real‐time qPCR

For cDNA synthesis, 50‐1000 ng of RNA were processed with Maxima First Strand cDNA Synthesis Kit (ThermoFisher). For the qPCR experiments, 1 µL of cDNA or plasmid templates were loaded into a 96‐well plate together with 10 µmol/L primer pairs and SYBR™ Green PCR Master Mix (ThermoFisher) and run using QuantStudio5 thermocycler (ThermoFisher). The primers used are listed in Table S1. For every assay, a reverse transcriptase negative control (*–rt*) with all primers used was run. In all tissues, the difference between the reference sample and the *–rt* mRNA was ≥8 cycles. Gene reference testing was carried out using dietary models and their respective controls. The two reference genes with the smallest difference between control and obese/T2DM models and with lowest standard deviation were chosen for the calculation of fold increase of IR (Table S2 and Figure S2).

### 2.5 Western Blot (WB)

30 mg of tissue, or 200 islets where homogenized using a tissue disruptor, the samples were frozen (−80°C) and thawed (4°C) 3 times and then centrifuged 11 000 g 30 minutes at 4°C. After denaturation, the samples were loaded onto 10% polyacrylamide gel stain‐free gel (Bio‐Rad, Hercules, CA, USA). Proteins were transferred onto nitrocellulose membrane using Bio‐Rad wet‐transfer apparatus (Bio‐Rad), imaged for the protein content using (ChemiDoc Imaging System, Bio‐Rad) and blocked for 1 hour at room temperature using Odyssey blocking buffer (Li‐Cor, Lincoln, NE, USA) or 5% milk powder in TBST. Membranes were incubated overnight with the appropriate primary antibodies in Odyssey blocking buffer or 5% BSA, washed and incubated 1 hour in the dark using secondary antibodies (Supporting information) and imaged using Odyssey imaging system (Li‐Cor) or ChemiDoc Imaging System (Bio‐Rad). WB data in tissue were normalized to the selected reference protein and total protein loading and then averaged. WB of isolated cells was normalized to total protein or DNA (data not shown).

### 2.6 Cell culture

The murine 3T3‐L1 MBX cell line was purchased from ATCC (Manassas, VA, USA). Culture and experimental conditions are described in Figure S4.

### 2.7 Adipose tissue fractionation

Perigonadal adipose tissue (pGAT) depots were dissected, washed 3 times with Krebs‐Ringer‐Phosphate (KRP) buffer, 1% BSA at 37°C for 10 minutes and incubated with 0.5 mg/mL collagenase type I (Sigma) in KRP 4% BSA at 37°C for 1 hour with shaking. The suspension was filtered twice through a screen size 60 mesh (Sigma) using KRP 0.1% BSA and centrifuged at 200 g, 4°C for 10 minutes. The fraction below the floating fraction was removed, fresh cold KRP 0.1% BSA was added and the cell suspension was centrifuged again. The floating fraction was retrieved, then TRIzol (ThermoFisher) was added and the samples were further processed for mRNA extraction. The residual stromal vascular fraction (SVF) was resuspended and processed using MACS LS columns and separators (Myltenyi Biotech, Bergisch Gladbach, Germany). To separate monocytes/macrophages, anti‐*f4/80* MicroBeads UltraPure (Myltenyi) were used. Both the separated fraction of monocytes/macrophages, called *f4/80+* cells and the SVF fraction, not retained in the column and called *f4/80−* cells, were centrifuged and 1 mL of TRIzol was added prior to mRNA extraction. To separate lymphocytes from the SVF *f4/80−* cells anti‐*cd3* MicroBeads UltraPure (Myltenyi) were used.

### 2.8 In situ hybridization

Organs were fixed in 10% neutral buffered formalin (Sigma). After 24 hours for liver, pancreas and brain, and 48 hours for pGAT, the tissues were placed in 70% ethanol prior to processing using a Tissue‐Tek VIP (Sakura Finetek, Alphen aan den Rijn, Nederland). Tissues were embedded in paraffin and cut into 4 μm sections using a microtome (Microm HM360, ThermoFisher). The sections were used for in situ hybridization using Base Scope^TM^ (ACDBio, Newark, CA, USA) paired double‐Z oligonucleotide probes. The IR‐B probe (BA‐Mm‐Insr‐tv2‐E11E12) targeting 2735‐2771 of NM 001330056.1 was visualized with red colour amplification. The IR‐A probe (BA‐Mm‐Insr‐tv1‐E10E11) targeting 2700‐2735 of NM 010568.3 was visualized in green. Negative (bacterial *dapb*) and positive (mouse *ppib* and *polr2a*) control probes were tested. Negative controls showed an almost negligible background (Figure S5B,D,E). Images were taken using a Leica SP8 equipped with a colour camera DFC7000T (Leica). Images were collected in Z‐stacks with 1.5 μm thickness. For each section, 1‐3 images were acquired using the navigator system on the LASX software, with maximum pixel resolution and using a 20× immersion objective. By using Fiji,^16^ each image was searched for red and green dots by eye and each dot was annotated using the Fiji cell counter plugin.

### 2.9 Statistics

IR calculations are explained in the Supporting information. Data representation, outliers and statistical analysis were performed using GraphPad Prism software (La Jolla, CA, USA). Depending on group's number and statistical distribution of the data, ANOVA, *T*‐Test, Mann‐Whitney or Kruskal‐Wallis tests together with Tukey or Dunn's post Hoc tests were used. Statistical significance was considered for *P* < .05 (*), < .01 (**), < .001 (***).

## 3 RESULTS

### 3.1 IR isoform mRNA expression in tissues of healthy and obese/T2DM mice

**FIGURE 1:**
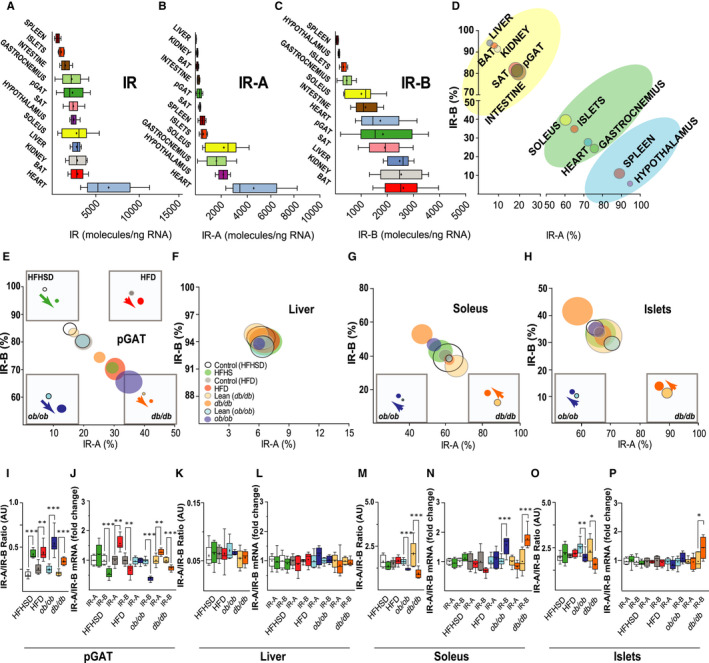
IR isoform gene expression in tissues of healthy mice and changes in obesity/T2DM. (A‐C) Amounts of IR (A), IR‐A (B) and IR‐B (C) as mRNA molecules per ng of RNA in different tissues measured by real‐time qPCR. Data are presented as mean, median and 10‐90 percentiles (n ≥ 8). IR molecules were calculated using the formula *x* = 10^(ct *^ *^k^* ^+^ *^b^* ^)^, where *k* is the slope (averages from IR‐A, IR‐B and IR Figure S1G,I,K,M) and *b* the intercept. The cDNA concentrations measured using PicoGreen^®^ was used to extrapolate the RNA amount per tissue. (D) Expression of IR isoform mRNA in tissues from C57BL/6J mice (as described in A‐C), calculated using ct values of IR‐A, IR‐B and IR from real‐time qPCR. Data are presented as confidence intervals (n = 12‐20). Tissues were divided in three categories depending on their prevalence of IR‐B (yellow), IR‐A (blue) or both (green). (E‐H) Percentages of IR isoform mRNAs in pGAT (E), liver (F), soleus (G) and pancreatic islets (H), calculated using ct values from real‐time qPCR and presented as confidence intervals. Insets show cohorts with significant differences, arrows indicate the direction of change. (I,K,M,O) IR‐A/IR‐B ratios in pGAT (I), liver (K), soleus (M) and pancreatic islets (O), calculated using ct values from real‐time qPCR and presented as mean, median and 10‐90 percentiles. (J,L,N,P) IR‐A and IR‐B gene expression in pGAT (J), liver (L), soleus (N) and pancreatic islets (L), normalized to tissue‐selected reference genes (Figure S2) and presented as fold change with mean, median and 10‐90 percentiles in comparison to the control for the specific cohort. Circles and boxes: white = control diet to HFHSD; green = HFHSD for 8 wk; grey = control diet to HFD; red = HFD for 14 wk; light blue = control to *ob/ob*; blue = *ob/ob* mice 3 mo old; light orange = control to *db/db*; orange = *db/db* mice 8 wk old. *P < .05, **P < .01, ***P < .001. Perigonadal adipose tissue (pGAT) n = 8; Liver n = 8; Soleus n = 8, Isolated pancreatic islets (islets) n ≥ 7

To quantitatively measure both the total amount of IR gene expression as well as the ratio of the IR isoforms, we established and validated a real‐time qPCR (Figure S1) and evaluated suitable reference genes for the different tissues (Figure S2 and Table S2). Applying this method, we first mapped the abundance of total IR mRNA as well as mRNAs of the two IR isoforms in a broad spectrum of tissues of C57BL/6J mice (Figure 1A‐D). With regard to IR isoform expression, the tissues can be divided into three distinct groups, one comprised of spleen and hypothalamus with high IR‐A and low IR‐B expression, a second group of adipose tissues, liver, kidney and intestine with high IR‐B and low IR‐A expression and a third group of muscle tissues and pancreatic islets with almost equal IR‐A and IR‐B expression (Figure 1B‐D).

To understand whether hyperglycemia and/or hyperinsulinemia affect the mRNA expression of total IR and/or the IR isoforms, we investigated a variety of tissues in genetic (*ob/ob* and *db/db*) and dietary (high‐fat high‐sucrose dietary [HFHSD] and high‐fat dietary [HFD]) mouse models of obesity, insulin resistance and T2DM. Among the ‘classical’ metabolic insulin target tissues, we found an increase in IR‐A/IR‐B ratio in perigonadal adipose tissue (pGAT) in all models studied (Figure 1E,I). The shift occurred in HFD and in *db/db* mice due to a decrease in IR‐B and an increase in IR‐A mRNA (Figure 1J), while in HFHSD and *ob/ob* mice the shift was solely due to a decrease in IR‐B mRNA expression. Similar to pGAT, in all models except HFD, brown adipose tissue (BAT) also displayed an increase in IR‐A/IR‐B ratio (Figure S3A,B). Subcutaneous adipose tissue (SAT) was the least affected of the adipose tissues, displaying only a trend towards the same changes observed in pGAT (Figure S3D‐F). In the liver, we did not find changes in IR isoform expression (Figure 1F,K,L) in any model. In skeletal muscle tissue, we found that IR isoform expression ratio was unaltered in the dietary mouse models (Figure 1G,M,N and Figure S3G‐I), in contrast in the genetic models we observed an increase in IR‐B which caused a decrease in the IR‐A/IR‐B ratio. In HFHSD mice, we found an increase in IR‐B and total IR in gastrocnemius (Figure S3G‐I), while in HFD, we saw no changes. The full data set with IR‐A and IR‐B mRNA percentages as well as IR‐A/IR‐B ratio in other organs such as heart, hypothalamus, kidney, intestine and spleen is shown in Table 1.

**TABLE 1:** IR isoform expression in mouse models of obesity/type 2 diabetes

Tissue	Model	Type	Number (n)	IR‐A (mean% ± CI)	IR‐B (mean% ± CI)	Ratio (IR‐A/IR‐B mean ± CI)	*P*‐value
pGAT	HFHSD	Control	8	15.5 ± 2.1	84.5 ± 2.1	0.184 ± 0.030	**<.001**
		HFHSD	8	29.3 ± 2.0	70.7 ± 2.0	0.417 ± 0.040	
	HFD	Chow	8	20.0 ± 3.0	80.0 ± 3.0	0.252 ± 0.049	**<.001**
		HFD	8	30.2 ± 2.9	70.3 ± 2.9	0.435 ± 0.072	
	*ob/ob*	Lean	8	19.5 ± 2.2	80.5 ± 2.2	0.244 ± 0.033	**<.001**
		*ob/ob*	8	34.8 ± 4.2	65.2 ± 4.2	0.539 ± 0.100	
	*db/db*	Lean	8	16.7 ± 1.6	83.3 ± 1.6	0.200 ± 0.023	**<.001**
		*db/db*	8	25.2 ± 1.9	74.8 ± 1.9	0.338 ± 0.034	
SAT	HFHSD	Control	8	20.2 ± 6.9	79.8 ± 6.9	0.266 ± 0.119	.19
		HFHSD	8	23.8 ± 2.4	76.2 ± 2.4	0.314 ± 0.042	
	HFD	Chow	8	16.1 ± 2.3	83.9 ± 2.3	0.193 ± 0.032	**<.001**
		HFD	8	24.0 ± 1.8	76.0 ± 1.8	0.317 ± 0.031	
	*ob/ob*	Lean	8	20.3 ± 2.8	79.7 ± 2.8	0.257 ± 0.042	.20
		*ob/ob*	8	22.1 ± 2.3	77.9 ± 2.3	0.285 ± 0.019	
	*db/db*	Lean	8	18.8 ± 2.8	81.2 ± 2.8	0.233 ± 0.044	**.019**
		*db/db*	8	22.3 ± 1.4	77.7 ± 1.4	0.288 ± 0.023	
Hypothalamus	HFHSD	Control	8	94.7 ± 0.6	5.3 ± 0.6	18.2 ± 2.0	**<.001**
		HFHSD	7	96.3 ± 0.3	3.7 ± 0.3	26.5 ± 2.1	
	HFD	Chow	6	94.6 ± 0.8	5.4 ± 0.8	18.0 ± 2.0	.31
		HFD	6	95.2 ± 0.7	4.8 ± 0.7	20.2 ± 2.4	
	*ob/ob*	Lean	8	95.6 ± 0.4	4.4 ± 0.4	21.9 ± 1.9	**.009**
		*ob/ob*	8	94.5 ± 0.9	5.5 ± 0.9	17.8 ± 2.5	
	*db/db*	Lean	4	91.7 ± 1.5	8.3 ± 1.5	11.2 ± 2.3	.48
		*db/db*	4	90.7 ± 2.7	9.3 ± 2.7	10.1 ± 3.0	
Heart	HFHSD	Control	8	72.6 ± 3.6	27.4 ± 3.6	2.73 ± 0.49	*.076*
		HFHSD	8	76.1 ± 2.0	23.9 ± 2.0	3.22 ± 0.35	
	HFD	Chow	6	71.5 ± 2.8	28.5 ± 2.8	2.53 ± 0.32	*.067*
		HFD	4	75.8 ± 6.1	24.2 ± 6.1	3.23 ± 1.44	
	*ob/ob*	Lean	8	70.4 ± 3.4	29.6 ± 3.4	2.44 ± 0.41	.55
		*ob/ob*	8	65.5 ± 2.4	34.5 ± 2.4	2.31 ± 0.27	
	*db/db*	Lean	8	71.7 ± 5.3	28.3 ± 5.3	2.69 ± 0.66	**.019**
		*db/db*	8	63.4 ± 5.4	36.6 ± 5.4	1.81 ± 0.43	
BAT	HFHSD	Control	8	6.4 ± 1.1	93.6 ± 1.1	0.068 ± 0.012	**<.001**
		HFHSD	8	12.0 ± 1.7	88.0 ± 1.7	0.136 ± 0.022	
	HFD	Chow	6	8.1 ± 1.6	91.9 ± 1.6	0.088 ± 0.013	.25
		HFD	5	9.6 ± 2.1	90.4 ± 2.1	0.106 ± 0.025	
	*ob/ob*	Lean	8	10.2 ± 1.6	89.8 ± 1.6	0.114 ± 0.021	**<.001**
		*ob/ob*	8	15.6 ± 0.8	84.4 ± 0.8	0.185 ± 0.011	
	*db/db*	Lean	7	7.6 ± 0.8	92.4 ± 0.8	0.083 ± 0.009	**<.001**
		*db/db*	8	17.6 ± 2.4	82.4 ± 2.4	0.215 ± 0.036	
Liver	HFHSD	Control	8	5.6 ± 2.3	94.4 ± 2.3	0.059 ± 0.015	.72
		HFHSD	8	5.9 ± 1.5	94.1 ± 1.5	0.063 ± 0.017	
	HFD	Chow	10	5.9.±0.7	94.1 ± 0.7	0.063 ± 0.008	.85
		HFD	10	5.9 ± 1.3	94.1 ± 1.3	0.063 ± 0.016	
	*ob/ob*	Lean	8	6.4 ± 1.1	93.6 ± 1.1	0.068 ± 0.023	.78
		*ob/ob*	7	6.0 ± 0.3	94.0 ± 0.3	0.064 ± 0,033	
	*db/db*	Lean	8	5.2 ± 1.1	94.8 ± 1.1	0.055 ± 0.013	.80
		*db/db*	8	5.3 ± 0.9	94.7 ± 0.9	0.057 ± 0.009	
Pancreatic islets	HFHSD	Control	7	64.6 ± 2.7	35.4 ± 2.7	1.84 ± 0.22	.61
		HFHSD	8	66.5 ± 5.3	33.5 ± 5.3	2.08 ± 0.47	
	HFD	Chow	4	66.3.±2.0	33.7 ± 2.0	1.97 ± 0.17	.34
		HFD	4	67.4 ± 3.7	32.6 ± 3.7	2.08 ± 0.34	
	*ob/ob*	Lean	8	70.6 ± 2.6	29.4 ± 2.6	2.43 ± 0.31	**.006**
		*ob/ob*	8	65.3 ± 2.6	34.7 ± 2.6	1.90 ± 0.24	
	*db/db*	Lean	7	67.6 ± 6.3	32.4 ± 6.3	2.21 ± 0.61	**.026**
		*db/db*	7	58.5 ± 5.3	41.5 ± 5.3	1.45 ± 0.29	
Soleus	HFHSD	Control	4	61.1 ± 8.3	38.9 ± 8.3	1.61 ± 0.58	.34
		HFHSD	4	57.1 ± 6.5	42.9 ± 6.5	1.35 ± 0.36	
	HFD	Chow	7	59.8.±3.6	40.2.±3.6	1.50 ± 0.23	.26
		HFD	8	61.8 ± 2.7	38.2 ± 2.7	1.65 ± 0.19	
	*ob/ob*	Lean	8	61.6 ± 1.8	38.4 ± 1.8	1.61 ± 0.12	**<.001**
		*ob/ob*	8	53.3 ± 3.8	46.7 ± 3.8	1.21 ± 0.10	
	*db/db*	Lean	8	65.8 ± 6.4	34.2 ± 6.4	2.06 ± 0.60	**<.001**
		*db/db*	8	46.9 ± 5.6	53.1 ± 5.6	0.91 ± 0.22	
Kidney	HFHSD	Control	8	7.7 ± 1.1	92.3 ± 1.1	0.083 ± 0.013	.74
		HFHSD	8	7.9 ± 0.8	92.1 ± 0.8	0.086 ± 0.010	
	HFD	Chow	8	9.3 ± 1.7	90.7 ± 1.7	0.104 ± 0.200	.29
		HFD	7	10.3 ± 1.2	89.7 ± 1.2	0.116 ± 0.015	
	*ob/ob*	Lean	8	14.4 ± 2.0	85.4 ± 2.0	0.168 ± 0.029	**.02**
		*ob/ob*	8	11.1 ± 1.9	88.9 ± 1.9	0.126 ± 0.024	
	*db/db*	Lean	7	7.7 ± 2.8	92.3 ± 2.8	0.084 ± 0.033	.61
		*db/db*	8	8.4 ± 1.1	91.6 ± 1.1	0.092 ± 0.013	
Spleen	HFHSD	Control	4	87.8 ± 7.0	12.2 ± 7.0	8.6 ± 8.5	.48
		HFHSD	4	88.9 ± 2.9	11.1 ± 2.9	8.2 ± 2.4	
	HFD	Chow	4	90.2 ± 3.2	9.8 ± 3.2	9.5 ± 2.9	.88
		HFD	4	90.6 ± 7.1	9.4 ± 7.1	11.8 ± 10.0	
	*ob/ob*	Lean	4	86.0 ± 6.5	14.0 ± 6.5	6.8 ± 5.6	.34
		*ob/ob*	4	82.8 ± 4.1	17.2 ± 4.1	4.9 ± 1.5	
	*db/db*	Lean	4	85.6 ± 4.8	14.4 ± 4.8	6.2 ± 3.0	.62
		*db/db*	4	84.2 ± 5.6	15.8 ± 5.6	5.6 ± 2.6	
Gastrocnemius	HFHSD	Control	8	75.0 ± 3.8	25.0 ± 3.8	3.13 ± 0.70	.22
		HFHSD	8	72.5 ± 3.1	27.5 ± 3.1	2.69 ± 0.42	
	HFD	Chow	8	76.0 ± 3.4	24.0 ± 3.4	3.26 ± 0.57	1.00
		HFD	7	75.8 ± 4.2	24.2 ± 4.2	3.26 ± 0.76	
	*ob/ob*	Lean	8	14.4 ± 2.0	85.6 ± 2.0	2.80 ± 0.51	**.008**
		*ob/ob*	8	11.1 ± 1.9	88.9 ± 1.9	2.02 ± 0.33	
	*db/db*	Lean	8	76.1 ± 4.6	23.9 ± 4.6	3.36 ± 0.75	**.001**
		*db/db*	8	62.5 ± 7.6	37.5 ± 7.6	1.81 ± 0.60	
Intestine	HFHSD	Control	4	18.1 ± 6.4	81.9 ± 6.4	0.22 ± 0.10	.20
		HFHSD	4	20.1 ± 7.3	79.9 ± 7.3	0.25 ± 0.13	
	HFD	Chow	4	20.6 ± 7.4	79.4 ± 7.4	0.26 ± 0.12	.34
		HFD	4	16.4 ± 5.9	83.6 ± 5.9	0.20 ± 0.08	
	*ob/ob*	Lean	4	21.3 ± 7.1	78.7 ± 7.1	0.27 ± 0.11	.88
		*ob/ob*	4	23.5 ± 0.6	76.5 ± 0.6	0.31 ± 0.10	
	*db/db*	Lean	4	18.6 ± 8.4	81.4 ± 8.4	0.23 ± 0.13	.82
		*db/db*	4	18.1 ± 3.6	81.9 ± 3.6	0.22 ± 0.05	

Abbreviations: BAT, brown adipose tissue; CI, confidence interval of the mean; HFD, high fat diet; HFHSD, high fat high sucrose diet; pGAT, perigonadal adipose tissue; SAT, subcutaneous adipose tissue.

*P*‐value: *P*‐values in bold represent statistical significant values compared to the control group of the model; *P*‐values in italic represent values below .1 but not statistically significant compared to the control group of the model.

### 3.2 Total IR mRNA expression and IR protein levels in ‘classical’ metabolic tissue in obesity/T2DM

**FIGURE 2:**
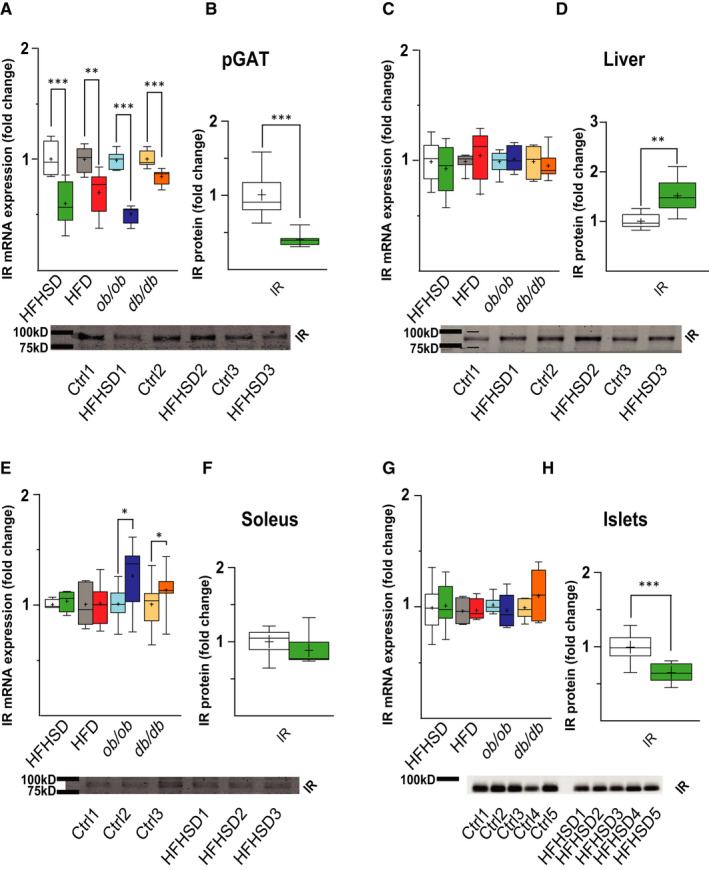
Total IR mRNA expression and IR protein levels in classical metabolic tissue in obesity/T2DM. (A,C,E,G) IR gene expression changes in pGAT (A), liver (C), soleus (E) and pancreatic islets (G), normalized to tissue‐selected reference genes (Figure S2) and presented as fold change with mean, median and 10‐90 percentiles in comparison with the control for the specific cohort. Boxes: white = control diet to HFHS; green = HFHSD for 8 wk; grey = control diet to HFD; red = HFD for 14 wk; light blue = control to *ob/ob*; blue = *ob/ob* mice 3 mo old; light orange = control to *db/db*; orange = *db/db* mice 8 wk old. pGAT n = 8; Liver n = 8; Soleus n = 8, Isolated pancreatic islets (islets) n ≥ 7. (B,D,F,H) Representative WB of IR and tissue‐selected normalizer after 8 wk of HFHSD in pGAT (B), liver (D), soleus (F) and pancreatic islets (H). Quantification via WB blot of IR data in pGAT (B) n = 7‐8, liver (D) n = 8, soleus (F) n = 7‐8 and pancreatic islets (H) n = 11‐13 normalized on the average of the tissue‐selected normalizer and total gel protein in pGAT, liver and soleus and α‐tubulin in islets (Figure S2). Boxes: white = control diet to HFHSD; green = HFHSD for 8 wk. *P < .05, **P < .01, ***P < .001 calculated using *T*‐Test

It is still unclear whether tissue IR mRNA levels correlate with total IR protein levels in obesity/T2DM. In tissues, mostly linked to glucose metabolism and insulin resistance, we found a significant decrease in total IR mRNA in pGAT (Figure 2A), which correlates with a decrease in total IR protein (Figure 2B). In liver, there were no changes in the total IR mRNA but an increase in IR protein (Figure 2C,D). In soleus muscle, we did not find changes in the total IR mRNA in dietary models but an increase in genetic models of obesity/T2DM (Figure 2E). In HFHSD, the absence of changes in IR mRNA was also reflected at the protein level (Figure 2F). In isolated pancreatic islets, we found no changes in mRNA levels in all models studied (Figure 2G). In contrast to liver and muscle tissue, in pancreatic islets, we found that in HFHSD the unchanged IR mRNA levels did not mirror the significant decrease of IR protein (Figure 2H).

In summary, while in some tissues the change in total, as well as in isoform‐specific IR expression, was more variable between the models, in pGAT, we found a consistent increase in the IR‐A/IR‐B ratio and a decrease in total IR mRNA levels in all obesity/T2DM models, which was mirrored in the decrease in IR protein.

### 3.3 Deconstructing tissue‐complexity of pGAT to investigate the change in cell‐type‐specific expression of IR isoforms

To understand the mechanisms involved in the change in tissue‐specific IR isoform expression, we decided to use pGAT for the following reasons. First, this tissue and its alteration during obesity/diabetes are well characterized^17^; second, we found consistent changes in IR/IR isoform expression in pGAT in all models; third, the changes in IR mRNA correlated with the IR protein levels; fourth, a similar increase in IR‐A was observed in human SAT after obesity/T2DM development.^10^

**FIGURE 3:**
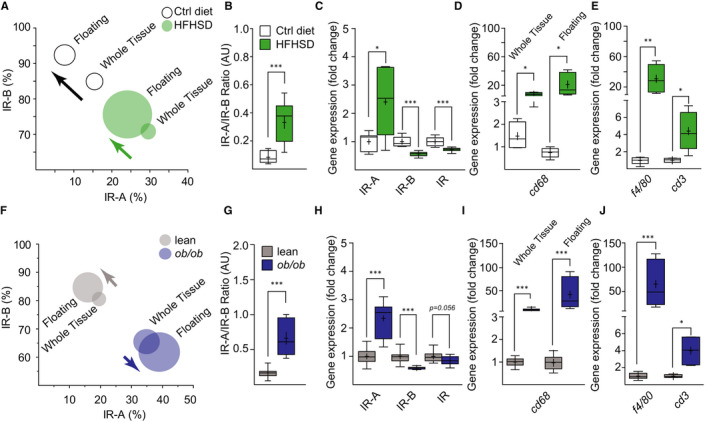
Deconstructing tissue‐complexity of pGAT to investigate cell‐type specific expression of IR isoforms by qPCR. (A) Percentages of IR isoform mRNAs in pGAT whole tissue and floating fraction from control diet and HFHSD, calculated using ct values from real‐time qPCR and presented as confidence intervals. Whole tissue n = 8; fractionated n = 9. (B) IR‐A/IR‐B ratio in pGAT floating fraction from control diet and HFHSD, calculated using ct values from real‐time qPCR and presented as mean, median and 10‐90 percentiles. (C) IR gene expression in pGAT floating fraction from control diet and HFHSD normalized to *tbp* and *hmbs* reference genes and presented as fold change with mean, median and10‐90 percentiles. Whole tissue n = 8; fractionated n = 9. (D) *cd68* gene expression in pGAT whole tissue and floating fraction from control diet and HFHSD normalized to *tbp* and *hmbs* reference genes. Data are presented as fold change with mean, median and 10‐90 percentiles. Whole tissue n = 4; fractionated n = 5. (E) *f4/80* and *cd3* gene expression in pGAT floating fraction from control diet and HFHSD normalized to reference gene *hmbs*. Data are presented as fold change with mean, median and 10‐90 percentiles, n = 5. (F) Percentages of IR isoform mRNAs in pGAT whole tissue and floating fraction from lean controls and *ob/ob* calculated using ct values from real‐time qPCR and presented as confidence intervals, n ≥ 8. (G) IR‐A/IR‐B ratio in pGAT floating fraction from lean controls and *ob/ob* calculated using ct values from real‐time qPCR and presented as mean, median and 10‐90 percentiles, n ≥ 8. (H) IR gene expression in pGAT floating fraction from lean controls and *ob/ob* normalized to reference gene *hmbs* and presented as fold change with mean, median and 10‐90 percentiles in comparison to the control diet, n ≥ 10. (I) *cd68* gene expression in pGAT whole tissue and floating fraction from lean controls and *ob/ob* normalized to reference gene *hmbs*. Data are presented as fold change with mean, median and 10‐90 percentiles, n = 4. (J) *f4/80* and *cd3* gene expression in pGAT floating fraction from lean controls and *ob/ob* normalized to reference gene *hmbs*. Data are presented as fold change with mean, median and 10‐90 percentiles, n ≥ 4. Circles and boxes: white = control diet to HFHSD; green = HFHSD for 8 wk; grey = lean controls to *ob/ob*; blue = *ob/ob* mice 3 mo old. Statistical significance was calculated using *T*‐Test or Mann‐Whitney depending on data distribution. * *P* < .05 ** *P* < .01 *** *P* < .001

We fractionated the pGAT from control and HFHSD mice and separated the floating fraction, which in control mice consists mainly of adipocytes, from the stromal vascular fraction. In the floating fraction of HFHSD mice, we found an increase in the IR‐A/IR‐B ratio, similar to the one found in whole pGAT (Figure 3A,B), and also a significant two‐fold increase in IR‐A (Figure 3C). The percentage of IR‐B in the floating fractions from both control and HFHSD mice was increased compared to the whole tissue, suggesting that IR‐B is the predominant form of IR in adipocytes. We additionally found a decrease in IR‐B and total IR mRNA in the floating fraction of pGAT from HFHSD mice (Figure 3C), similar to the changes in the whole tissue. However, since the floating fraction from obese mice is known to contain also immune cells,^18^, ^19^ we investigated the presence of immune cells in this fraction by measuring monocyte/macrophage gene markers. As expected, in the whole pGAT and in its floating fraction of HFHSD mice we found an increase in *cd68* and *f4/80* mRNA (Figure 3D,E), indicating an enrichment of these immune cells. Moreover, we found an over four‐fold increase of the lymphocyte marker *cd3* in HFHSD‐treated mice compared to their controls (Figure 3E). To test whether this is a common feature in obesity, we analysed the same parameters in pGAT from 3‐months‐old *ob/ob* mice and obtained similar results (Figure 3F‐J).

**FIGURE 4:**
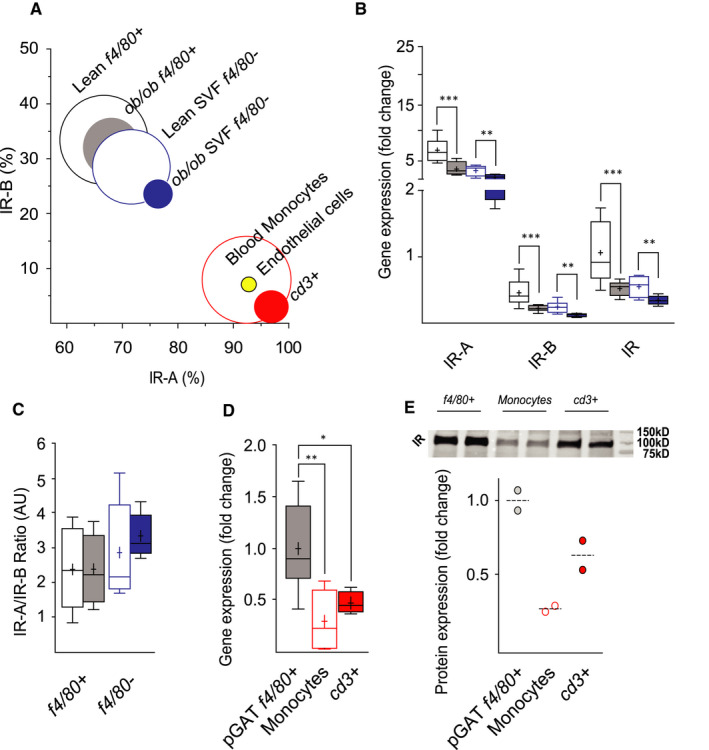
Identify changes in total IR and IR isoform expression in the specific cell types that form pGAT. (A) Percentages (CI) of IR isoform mRNAs in *f4/80+* and *f4/80−* cells of the stromal vascular fraction (SVF) from *ob/ob* mice and lean littermates (n ≥ 8), blood monocytes (n = 4), *cd3*+ cells (n = 3) and isolated endothelial cells (n = 3) from adult C57BL/6J mice, calculated using ct values from real‐time qPCR and presented as confidence intervals. Circles: Black empty = *f4/80+* cells from lean pGAT; grey = *f4/80+* cells from *ob/ob* pGAT; blue empty = *f4/80−* cells from lean pGAT; blue = *f4/80−* cells from *ob/ob* pGAT; red empty = C57BL/6J blood circulating monocytes, yellow = isolated endothelial cells, red = *cd3*+ cells. (B) IR gene expression in *f4/80+* and *f4/80−* cells of the SVF normalized to reference gene *hmbs* and presented as fold change with mean, median and 10‐90 percentiles in comparison with the floating fraction IR expression of control mice, n ≥ 8. Boxes: black empty = lean *f4/80+* cells; grey = *ob/ob f4/80+* cells; blue empty = lean *f4/80−* cells; blue = *ob/ob f4/80−* cells. (C) IR‐A/IR‐B ratio in *f4/80+* and *f4/80−* cells from pGAT of *ob/ob* and lean animals calculated using ct values from real‐time qPCR and presented as mean, median and 10‐90 percentiles, n ≥ 8. Boxes: Black empty = *f4/80+* cells from lean pGAT; grey = *f4/80+* cells from *ob/ob* pGAT; blue empty = *f4/80−* cells from lean pGAT; blue = *f4/80−* cells from *ob/ob* pGAT. (D) IR gene expression in *f4/80+* cells from lean pGAT (grey), C57BL/6J blood circulating monocytes (empty red) and *cd3*+ (filled red) cells normalized to reference gene *hmbs* and presented as fold change compared to pGAT *f4/80+* with mean, median and 10‐90 percentiles, n ≥ 4. Statistical significance was calculated using *T*‐Test or Mann‐Whitney depending on data distribution. *P < .05 **P < .01 ***P < .001. (E) IR protein expression in *f4/80+* cells from C57BL/6J mice (grey), blood circulating monocytes (empty red) and pGAT *cd3*+ (filled red) cells normalized to total protein and presented as fold change compared to compared to pGAT *f4/80+* cells. Every circle represents a pool of 5 mice

Our findings indicate that the shift in IR isoform expression in pGAT of obese/T2DM mice could be a consequence of the change in tissue composition/architecture rather than a change in IR pre‐mRNA splicing in adipocytes. In fact, in the widely accepted adipocyte murine cell model 3T3‐L1, in which the IR isoform expression is similar to primary adipocytes, treatment with 10 nmol/L insulin or 5 ng/mL TNFα did not affect the isoform expression pattern at the mRNA level (Figure S4). We used pGAT of *ob/ob* mice to characterize the non‐adipocytes present in the tissue. After removing the floating fraction from pGAT, we isolated monocytes/macrophages (*f4/80+* cells) from the stromal vascular fraction (SVF), composed of other non‐ monocytes/macrophages immune cell types, fibroblasts, endothelial cells and pre‐adipocytes. In both *f4/80+* and *f4/80−* fractions of the SVF, we found that the majority of IR mRNA was IR‐A (Figure 4A‐C). Comparison between lean and obese mice *f4/80+* and *f4/80−* fractions showed a decrease in total IR mRNA expression (Figure 4B) but no change in IR isoform ratio (Figure 4C). The decrease in total IR mRNA in *f4/80+* and *f4/80−* fractions and in the whole pGAT could be due to cells recruited from the blood circulation, which might express less IR mRNA. In fact, we found that blood monocytes express almost exclusively IR‐A (Figure 4A) and a lower amount of total IR mRNA, compared to the *f4/80+* cell population in pGAT (Figure 4D), consisting of resident macrophages and infiltrating monocytes/macrophages. Moreover, blood lymphocytes (*cd3+* cells), which we found increased in pGAT of HFHSD and *ob/ob* mice and can be found in the *f4/80−* fraction, also express mostly IR‐A and have lower IR mRNA and protein expression compared to pGAT *f4/80+* cells (Figure 4A,D,E).

In summary, we found that resident/infiltrating macrophages, monocytes and lymphocytes as well as other cell types, which are known to increase in magnitude in pGAT during obesity, express predominantly IR‐A. Moreover, infiltrating monocytes and lymphocytes express less total IR mRNA and protein, compared to tissue‐resident macrophages of pGAT, thus decreasing the total IR mRNA and IR protein of the tissue. The infiltrating cell populations increased the IR‐A/IR‐B ratio, decreased the IR‐B and the total IR in the whole pGAT tissue.

### 3.4 Visualizing the IR isoform mRNA in situ at cellular resolution in the context of pGAT histology

**FIGURE 5:**
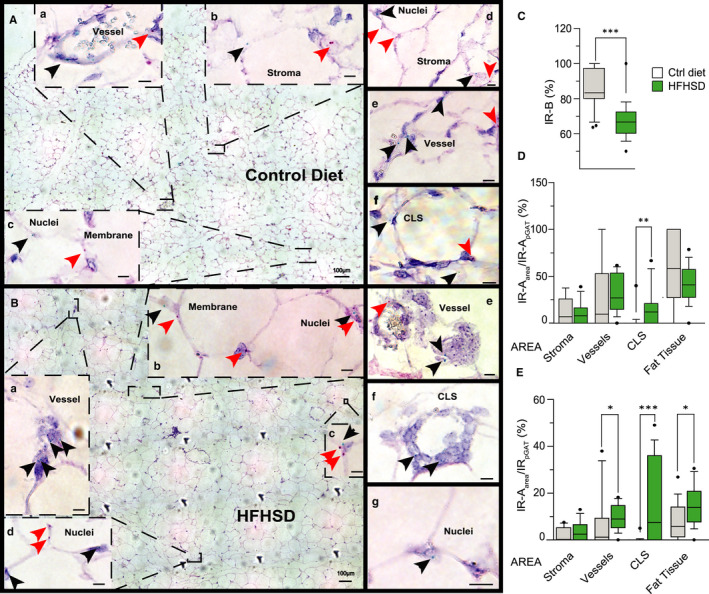
Visualizing the IR isoform mRNA in situ at cellular resolution in the context of pGAT histology. (A) Representative image of in situ hybridization of pGAT of a control diet mouse. Arrowheads in blow‐ups indicate single mRNA molecules. IR‐A green dots are indicated with black arrowheads, while IR‐B red dots are indicated with red arrowheads. (a‐f) Representative images of in situ hybridization in pGAT of a control diet mouse, showing IR‐A (black arrowheads) and IR‐B (red arrowheads) localization in the categories *fat tissue* (membranes and nuclei within the parenchyma), *stroma*, *vessels* and *CLS* in control diet mice. (B) Representative image of in situ hybridization of pGAT of a HFHSD mouse. Arrowheads in blow‐ups indicate single mRNA molecules. IR‐A green dots are indicated with black arrowheads, while IR‐B red dots are indicated with red arrowheads. (a‐g) Representative images of in situ hybridization of pGAT of a HFHSD mouse, showing IR‐A (black arrowheads) and IR‐B (red arrowheads) localization in the categories *fat tissue* (membranes and nuclei within the parenchyma), *stroma*, *vessels* and *CLS*. (A,B) Scale bar: 100 µm; (a‐g) 10 µm. For the pGAT localization of IR‐A mRNA after screening the large tissue image for green dots (representing IR‐A), another more specific image was taken to verify the presence of the IR‐A dot. (C) Percentage of IR‐B identified by in situ hybridization in pGAT of control diet (grey boxes) and HFHSD (green boxes) mice. Each box presents median and 10‐90 percentile of at least 19 analysed images from 3 animals. Black circles represent outliers, which were not omitted from the *T*‐Test analysis. ***P < .001. (D) Percentage of IR‐A related to total IR‐A in specific pGAT areas assigned to different categories called: *fat tissue*, where the dots where identified in cells distant more than one cell away from vessels or stroma, *vessels* (within the vessel or in the close proximity to the vessel), *stroma* (cells distant maximum one cell away from the stroma) or *crown‐like‐structures (CLS)* (within the cells composing the CLS). The percentage of each IR isoform was than calculated for each large image. Control (grey boxes) and HFHSD (green boxes) mice. Each box is represented by median and 10‐90 percentile of at least 18 analysed images from 3 animals. Black circles represent outliers, which were not omitted from the *T*‐Test analysis or Mann‐Whitney, depending on data distribution, **P < .01. (E) Percentage of IR‐A related to total IR in specific pGAT areas categorized as *stroma*, *vessels*, *crown‐like structures (CLS)*, or *fat tissue* of control (grey boxes) and HFHSD (green boxes) mice. Each box presents median and 10‐90 percentile of at least 18 analysed images from 3 animals. Black circles represent outliers, which were not omitted from the *T*‐Test analysis or Mann‐Whitney depending on the data distribution, **P < .01

To confirm that the infiltration of immune cells leads to the increase in IR‐A/IR‐B ratio in pGAT, we decided to investigate the IR isoform expression directly in the tissue. Due to the lack of IR isoform‐specific antibodies, we used the BaseScope mRNA in situ hybridization technique to visualize IR isoform mRNA molecules at single‐cell level. After binding either to the exon 11/12 junction (IR‐B mRNA) or exon 10/12 junction (IR‐A mRNA), the probes were amplified by double colorimetric amplification in the same section and were detected as individual coloured dots. Analysing IR isoform expression in liver, pancreatic islets and pGAT of control animals, we found that the IR‐A/IR‐B ratio was comparable to the one detected by real‐time qPCR (Figures 5A and S5F), thus demonstrating that this approach is specific and selective for the individual mRNA isoforms. Moreover, comparing IR‐A/IR‐B ratio in pGAT from mice, fed either control diet or HFHSD, we got similar results to the ones obtained by real‐time qPCR analysis (Figure 5A‐C).

To further clarify which cells are responsible for an increase in IR‐A/IR‐B ratio in pGAT in obese animals, we analysed the localization of each green IR‐A dot in sections of control diet and HFHSD fed mice. In HFHSD fed mice, we found a significant increase of IR‐A mRNA dots in crown‐like structures (*CLS*), which were barely detectable in control diet mice, and a smaller but significant increase in IR‐A mRNA in the categories *vessel* and *fat tissue* (Figures 5D,E and S5). The latter category includes adipocytes but also other cell types with nuclear shape not consistent with that of classical adipocytes. In this category, the increase in the IR‐A/IR ratio in HFHSD mice (Figure 5E) is approximately three‐fold lower compared to the observed increase in IR‐A expression in the whole pGAT (Figure 3A). This again indicates that the changes in IR isoform expression pattern in pGAT is mainly due to the presence of immune cells expressing mainly IR‐A, which infiltrate the tissue from the vessels and generate CLS.

## 4 DISCUSSION

Understanding the expression pattern of total IR as well as of IR isoforms in tissues in health and disease is a prerequisite for IR‐targeted therapies. The two IR isoforms IR‐A and IR‐B exhibit different tissue distribution depending on the species.^5^, ^19^, ^20^, ^21^ After ligand binding, IR isoforms transduce different signals by employing different signalling cascades in pancreatic β‐cells, where both receptor isoforms are co‐expressed.^7^, ^8^, ^9^ Whether this is the case also in other cell types remains an unsolved question. In addition to insulin, IR‐A displays a high affinity for IGF2 and proinsulin, and low affinity for IGF1.^2^, ^22^ An increase in IR‐A could thus enhance IGF2‐mediated mitogenic signalling and decrease metabolic insulin signalling, which has been suggested as a cause of insulin resistance during the progression of diabetes.^14^, ^19^, ^23^, ^24^, ^25^ However, whether and how development of obesity/T2DM affects the IR isoform expression pattern in various tissues of humans has barely been explored^10^, ^12^, ^25^, ^26^ and even less in common animal models used in research. In order to identify changes in IR isoform expression in obesity/T2DM, we generated a novel data set of tissue‐specific IR gene expression in both genetic (*ob/ob*, *db/db*) and dietary (HFD, HFHSD) mouse models of obesity/T2DM and their healthy controls. Our most consistent finding was a change in the IR‐A/IR‐B ratio in pGAT. An increase in IR‐B was found in several tissues (such as muscle, heart and pancreatic islets) in the genetic models (*db/db* and *ob/ob*) and is probably linked to the severe hyperglycaemia/hyperinsulinemia in these mice. In fact, other reports showed an increase in IR‐B in pancreatic β‐cells and pancreatic islets after exposure to extreme hyperglycaemic and hyperinsulinemic conditions.^27^, ^28^ Interestingly, while total IR gene expression and IR‐A/IR‐B ratio was altered in pGAT and can be potentially linked to insulin resistance, there were no consistent changes in muscle and liver.

In the dietary model HFHSD, which resembles best the western‐style fast food consumption, we compared the total IR mRNA to the total IR protein levels to understand their correlation. In the organs, mostly responsible for glucose metabolism such as adipose tissue, liver, muscle and islets, we show that changes in total IR gene expression in obesity/T2DM at the mRNA level are not always mirrored at the IR protein level. This suggests that the IR life cycle in different tissues is differently regulated and it is consequently pivotal to study individual tissues to be able to resolve the mechanisms underlying a change in IR expression pattern in disease.

To understand the mechanisms that underlie the changed IR expression pattern, we choose to investigate pGAT. In this tissue, the IR‐A/IR‐B ratio was increased in all four mouse models we studied. A similar change was found in humans during disease^10^ and the alterations during obesity/diabetes are well studied and characterized by an inflammatory response and infiltration of immune cells.^17^, ^29^ Although an increase in the IR‐A/IR‐B ratio in human adipose tissue in obesity was linked to changes in splicing factors,^10^ a change in the studied parameters (including splicing factors, mRNA expression of IR, etc) could also be a result of changes in the tissue composition. As shown before elsewhere,^18^, ^29^ in the floating fraction from pGAT we also found, beside adipocytes, other cell types known to infiltrate the adipose tissue in obesity. Lipid‐rich macrophages, which are a result of uptake of necrotic fat cells, have been described to accumulate in inflamed adipose tissue.^30^, ^31^ We found that tissue‐resident macrophages in control mice express a similar total IR mRNA amount as adipocytes, but predominantly the IR‐A type. T‐lymphocytes were also found in the floating fraction following fat separation and they also mostly express IR‐A. This led us to speculate that non‐adipocyte cells could be the main source responsible for the increase in IR‐A/IR‐B ratio. In fact, we did not find a change in IR isoform expression in a commonly used adipocyte model in vitro in response to hyperinsulinemia or inflammatory stimuli. It is worth noting that lymphocytes and monocytes, which express almost exclusively IR‐A, express less total IR mRNA and protein than pGAT‐resident macrophages. This might explain the relative decrease in total IR in pGAT in obesity, the magnitude of which is in line with the changes in cell subpopulations in pGAT in obesity in mice,^17^ favouring the hypothesis that adipocytes do not change their IR isoform or total IR gene expression. A similar mechanism with different dynamics might be the explanation for the similar trend in increase of IR‐A/IR‐B observed in BAT and SAT. The fact that in adipose tissue the total IR mRNA amount has been inversely correlated with body weight^32^ might be a consequence of infiltrating immune or other SVF cells into the inflamed tissue.

Analysis of IR isoform mRNAs in homogenized tissues using a real‐time qPCR approach is more quantitative, compared to previous densitometric estimation of PCR products,^21^ but it does not allow resolution at single‐cell level. To be able to resolve the IR isoforms in situ for the first time, we applied a novel hybridization technique that allowed us to identify the IR isoform mRNAs at cellular resolution without removing the cells from their tissue context, thus providing a significant methodological advance. Moreover, the two IR isoforms can be visualized as dual colours in the same sample, avoiding variation in probe hybridization due to the different quality of mRNA in different sections. With this method, we reproduced the numerical ratio of our real‐time qPCR data with the advantage of getting information on the localization of IR isoform mRNA in specific cells. By studying the localization of the IR‐A mRNA, we show that in control mice, the few IR‐A mRNA molecules are distributed between the parenchymal cells, cells along the blood vessel, close to the stroma and the few crown‐like structures (CLS) detected, the latter being a cluster of macrophages and other immune cells. In HFHSD fed obese mice, we found high amounts of CLS and IR‐A within those cells, but also a significant increase of IR‐A in cells along or close to the vessels. We link this observation to immune cells exiting the vessels and infiltrating the adipose tissue. These cell types are the main source responsible for the increase in the IR‐A/IR‐B ratio in pGAT in obesity/T2DM. Beside CLS and cells in proximity of vessels, we found a small but significant increase of IR‐A within the pGAT tissue parenchyma, which could include adipocytes but probably consists of other cell types, since often the shape of the nuclei was not consistent with that of classical adipocytes. Considering the magnitude of the shift in IR isoform expression, these areas are unlikely to play a major role for the significant increase in IR‐A/IR‐B ratio observed in the whole tissue.

We are fully aware that IR gene transcription and pre‐mRNA splicing are not the only levels that regulate IR isoform availability and function. In fact, it will be of utmost importance to understand the dynamics of IR isoform proteins at the cell surface, where they initiate signal transduction. Unfortunately, there are currently no tools available for the IR isoforms to tackle this question. Moreover, even though data from human subcutaneous fat, liver and pancreatic islets point towards a similar IR‐A/IR‐B mRNA ratio comparable to mice,^10^, ^11^, ^21^, ^27^, ^28^, ^32^ further studies are necessary by applying the here described approach to uncover the tissue‐specific mechanisms of changes in IR isoform mRNA expression in human pathology. In this context, our work provides a first step towards the understanding of the physiology and regulation of IR isoform expression in complex tissues. First, we provide an informative dataset of total IR‐ and IR isoform gene expression in a broad spectrum of tissues in health and in obesity/diabetes valuable to understand the IR regulation at transcriptional level. Second, we provide an important methodological advance that allows the analysis of IR isoform mRNA at cellular resolution. Third, we added further information on the IR isoforms in leucocyte subpopulations. Finally, exemplified on pGAT, we provide a mechanism for the change in IR isoform expression pattern in adipose tissue in obesity/T2DM.

## CONFLICTS OF INTEREST

P‐OB is cofounder and CEO of Biocrine AB, IBL and BL are consultants for Biocrine AB.

## AUTHOR CONTRIBUTIONS

**Noah Moruzzi:** Conceptualization (equal); Data curation (lead); Formal analysis (lead); Funding acquisition (supporting); Investigation (lead); Methodology (lead); Validation (lead); Visualization (lead); Writing‐original draft (lead); Writing‐review & editing (equal). **Francesca Lazzeri‐Barcelo:** Investigation (supporting); Methodology (supporting); Visualization (supporting); Writing‐review & editing (equal). **Ismael Valladolid‐Acebes:** Investigation (supporting); Methodology (supporting); Resources (supporting); Validation (supporting); Writing‐review & editing (supporting). **Tilo Moede:** Investigation (supporting); Methodology (supporting); Visualization (supporting); Writing‐review & editing (supporting). **Meike Paschen:** Investigation (supporting); Resources (supporting); Writing‐review & editing (supporting). **Barbara Leibiger:** Conceptualization (equal); Data curation (supporting); Formal analysis (supporting); Investigation (supporting); Methodology (supporting); Validation (supporting); Visualization (supporting); Writing‐original draft (equal); Writing‐review & editing (equal). **Per‐Olof Berggren:** Conceptualization (supporting); Funding acquisition (lead); Resources (equal); Writing‐original draft (equal); Writing‐review & editing (equal). **Ingo B. Leibiger:** Conceptualization (equal); Funding acquisition (supporting); Investigation (supporting); Methodology (supporting); Project administration (lead); Supervision (lead); Writing‐original draft (equal); Writing‐review & editing (equal).

## Supporting information

Supplementary Material

Figure S1

Figure S2

Figure S3

Figure S4

Figure S5

## Data Availability

The data that support the findings of this study are available from the corresponding author upon reasonable request.
