# Supplementary material for: Tissue‐specific expression of insulin receptor isoforms in obesity/type 2 diabetes mouse models
Source: J Cell Mol Med. 2021 Mar 19;25(10):4800–13. doi: 10.1111/jcmm.16452 (PMC8107091; doi:10.1111/jcmm.16452)
Supplement: Supplementary file 1 — Supplementary Material [file JCMM-25-4800-s004.pdf]

Figure S1

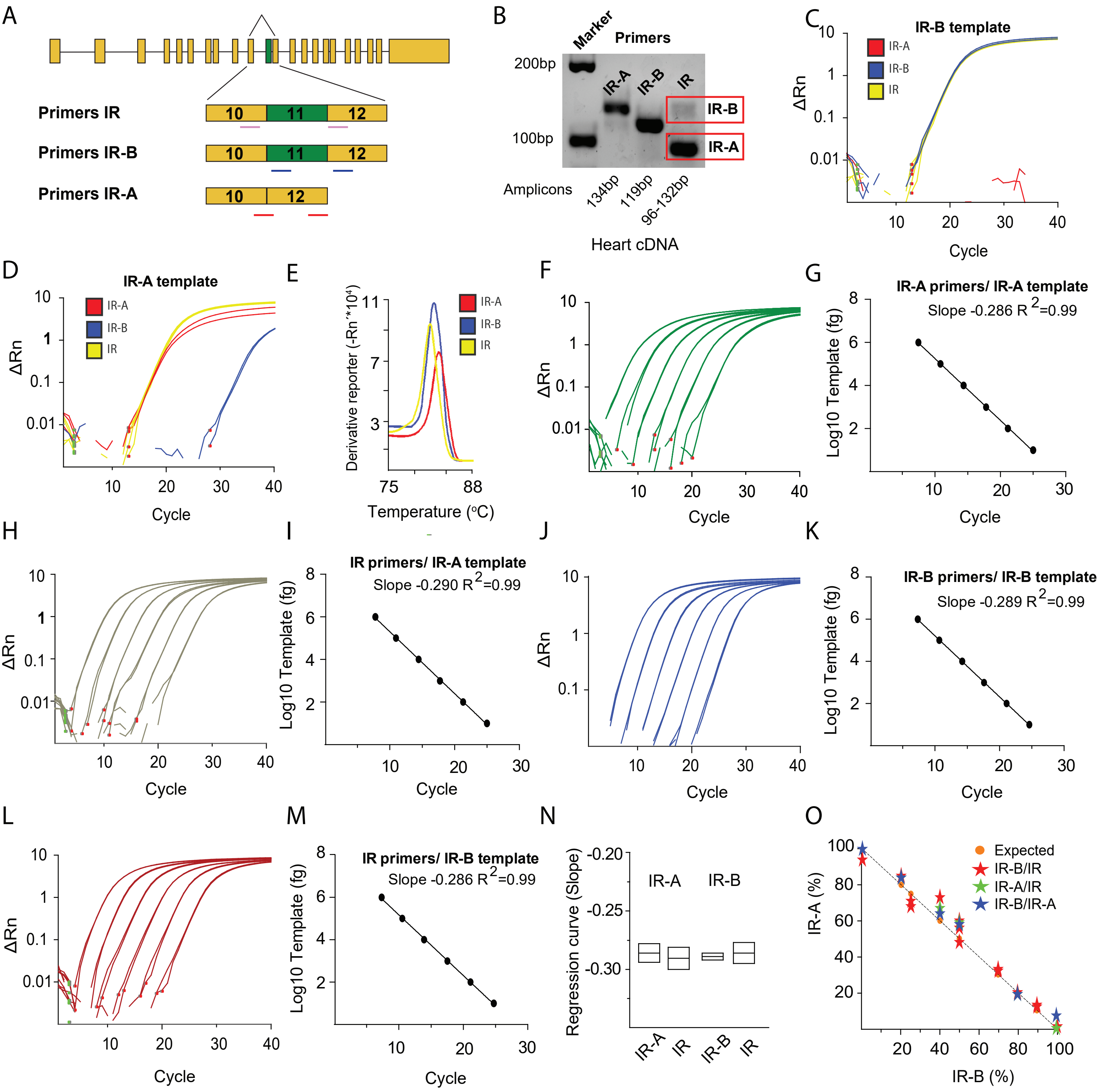

Primer specificity and selectivity for detection of total and isoform specific IR gene expression using real time qPCR.
Primer pairs for IR mRNA, amplifying both IR-A and IR-B, were designed to bind upstream and downstream of exon 11; for IR-B mRNA the forward primer binds to exon 11; for IR-A mRNA, the forward primer binds to the exon 10-12 junction **(A)**. The primers´ specificity was confirmed using mouse tissue cDNA by agarose gel electrophoresis **(B)** and melting curve analysis **(E)**. IR-B primers did not amplify IR-A template, while IR-A primers weakly amplified the IR-B template with a delay of 16 cycles **(C-D)**. Regression curves and slope calculation for each primer/template pair was carried out using the plasmids expressing IR-A and IR-B amplicons serially diluted from 1ng to 10fg **(F-M)**. To compare and standardize the calculations of IR isoforms using the ct value, an equal threshold for all three curves was chosen. The efficiency calculated for the IR-A plasmid (mean±CI for IR-A primers 93.5±2.5%; IR primers 95±4%) and the IR-B plasmid (mean±CI for IR-B primers 94.5±1.5%; IR primers 93±4%) as well as the regression slopes for each primer/template pair **(N)** did not differ among primers. We prepared different mixtures of IR-A and IR-B plasmids, containing between 0 and 100% of them respectively **(O)**. Using specific primers for the IR isoforms as well as for total IR, we measured the percentage of IR isoform amplicons amplified from the plasmid mixtures using the ct values of IR, IR-A and/or IR-B and compared the results to the predicted values (percentage of molecules used in the dilutions). The results were comparable to the expected values derived from the plasmid mixtures.
(A) Scheme of IR gene, exons (yellow and green) and the location of primers designed to recognize IR (both IR-A and IR-B; pink lines), IR-B (blue lines) and IR-A (red lines). Exon 11, which is present in IR-B and absent in IR-A, is shown in green. **(B)** Image of amplicons from real-time qPCR, performed in heart tissue of control mice, separated on a 3% agarose gel. IR primers amplify both IR isoforms (product size IR-B 132bp; IR-A 96bp; highlighted in the red rectangles). IR-A specific primers amplify the IR-A amplicon (134bp) and IR-B specific primers amplify the IR-B amplicon (119bp). **(C,D)** Real-time qPCR amplification curves using template plasmids containing the cDNA of amplicons generated using either IR-B or IR-A primers. Primers specific for IR-A (red), IR-B (blue) and IR (yellow) were used for amplification. **(E)** Melting temperatures of qPCR-products amplified from mouse tissue using primers for IR-A (red), IR-B (blue) and IR (yellow). **(F-M)** Titration of plasmids templates containing IR-A (F-I) or IR-B (J-M) amplicons using real-time qPCR and IR-A (F,G), IR-B (J,K) or IR (H,I,L,M) specific primers. Template concentration ranged from 10fg to 1ng. The regression curves and slopes were calculated by fitting the ct values of the average of duplicate measurements for IR-A primers on IR-A amplicon-containing plasmid template (G), IR-B primers on IR-B amplicon-containing plasmid template (K) and IR primers on IR-A (I) and IR-B (M) amplicon-containing plasmid templates. **(N)** Representation of mean slope and confidence interval of regression curves (n=2) generated using IR-A and IR-B amplicon-containing plasmid templates and IR primers. **(O)** Percentage of IR isoform amplicons (colored stars) obtained from a known mixture of IR-A and IR-B amplicon-containing plasmid templates (orange circles). The percentage was calculated using IR-B and IR ct values (red), IR-A and IR (green) or IR-B and IR-A (blue) as described in the method section.
The dotted line represents the expected ratio.

Figure S2

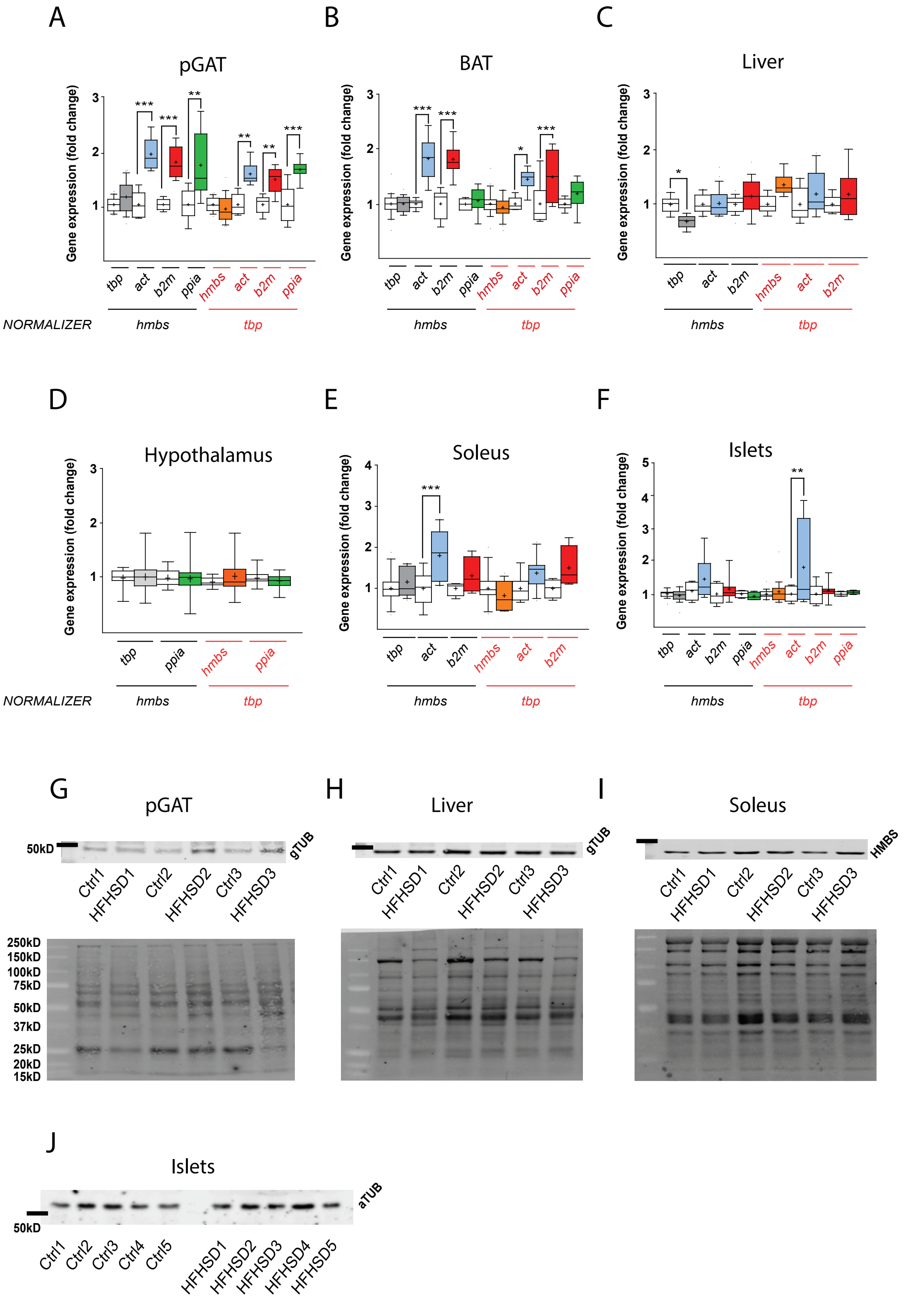

Assessment of tissue-specific reference genes and Western blot membrane protein/ normalizer for Figure 2.
(A-F) Quantification of real-time qPCR data of candidate reference genes normalized to either *hmbs* (black) or *tbp* (red) in control diet mice compared to dietary treatments (HFHSD and HFD combined). Data are presented as fold change mean, median and 10-90 percentile compared to control cohorts (black empty boxes). Boxes: grey = *tbp*; blue = *actin*; red = *b2m*; green = *ppia*; orange = *hmbs*. Statistical significance was calculated using one-way ANOVA and Bonferroni´s post test. Minimum n for each tissue: (A) perigonadal adipose tissue (pGAT) n=6; (B) brown adipose tissue (BAT) n=6; (C) liver n=9; (D) hypothalamus n=6; (E) soleus n=4; (F) isolated pancreatic islets (islets) n=7, * $p<0.05$ ** $p<0.01$ *** $p<0.001$.
The chosen reference genes for each tissue are bolded in Table S2.
(G-J) Representative blot of tissue selected normalizer and protein transferred used to normalize the IR in Figure 2. pGAT (G), liver (H), soleus (I) and pancreatic islets (J).

Figure S3

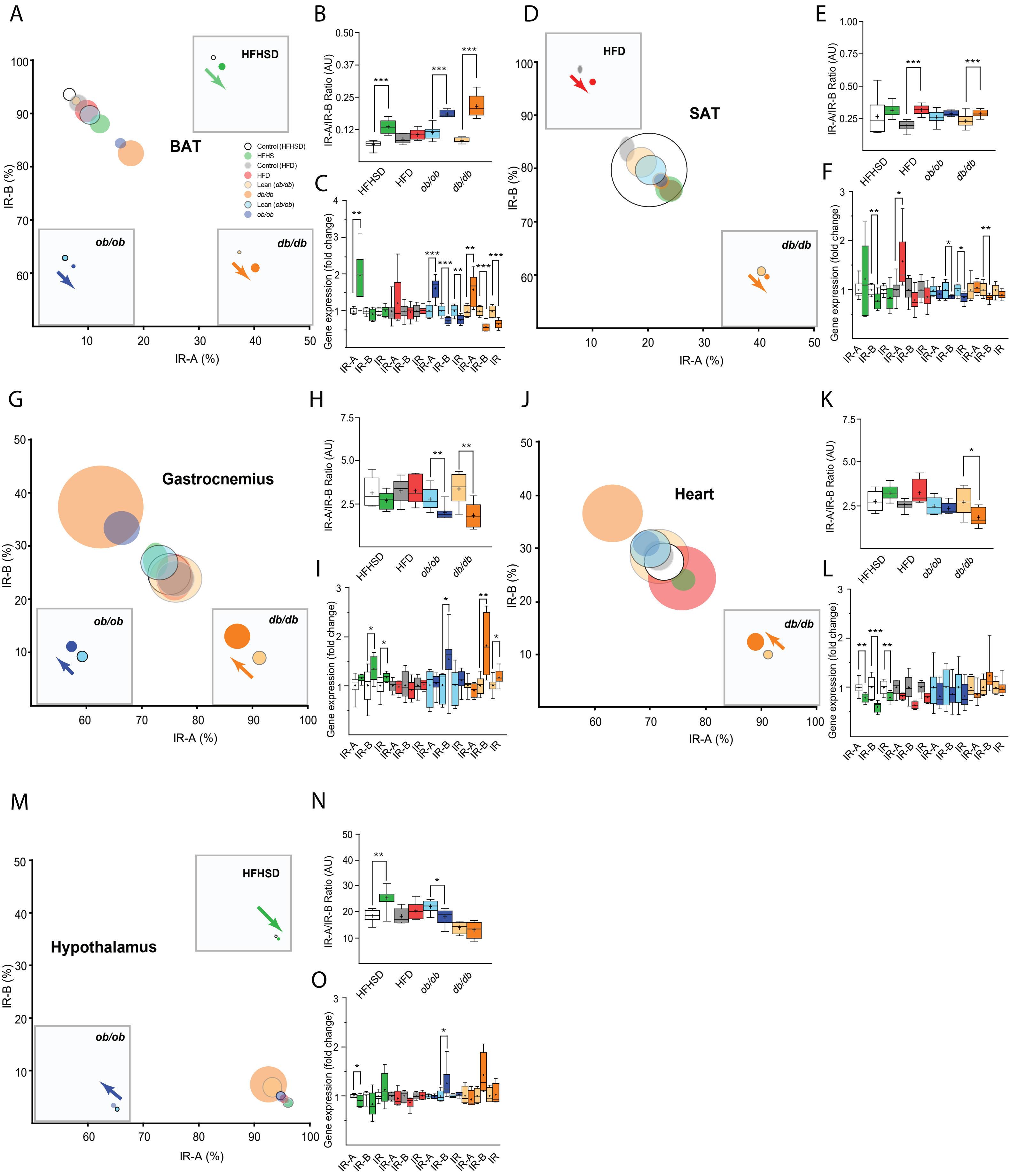

Tissue-specific changes of IR mRNA and its isoforms in obesity/T2DM

(A,D,G,J,M) Percentages of IR isoform mRNAs in brown adipose tissue (BAT) (A), subcutaneous adipose tissue (SAT) (D), gastrocnemius (G), heart (J), and hypothalamus (M) calculated using ct values from real-time qPCR and presented as confidence intervals. Insets show cohorts with significant differences, arrows indicate the direction of change. **(B,E,H,K,N)** IR-A/IR-B ratio in BAT (B), SAT (E), gastrocnemius (H), heart (K), and hypothalamus (N), calculated using ct values from real-time qPCR and presented as mean, median and 10-90 percentiles. **(C,F,I,L,O)** IR gene expression in BAT (C), SAT (F), gastrocnemius (I), heart (L), and hypothalamus (O), normalized to tissue-selected reference genes (Figure S2) and presented as fold change with mean, median and 10-90 percentiles in comparison to the control for the specific cohort.

Circles and boxes: black empty = control diet to HFHS; green = HFHSD for 8 weeks; gray = control diet to HFD; red = HFD for 14 weeks; light blue = control to *ob/ob*; blue = *ob/ob* mice 3 months old; light orange = control to *db/db*; orange = *db/db* mice 8 weeks old. * p<0.05 ** p<0.01 *** p<0.001. BAT n≥5; SAT n=8; Gastrocnemius n≥7; Heart n≥4, Hypothalamus n≥4.

Figure S4

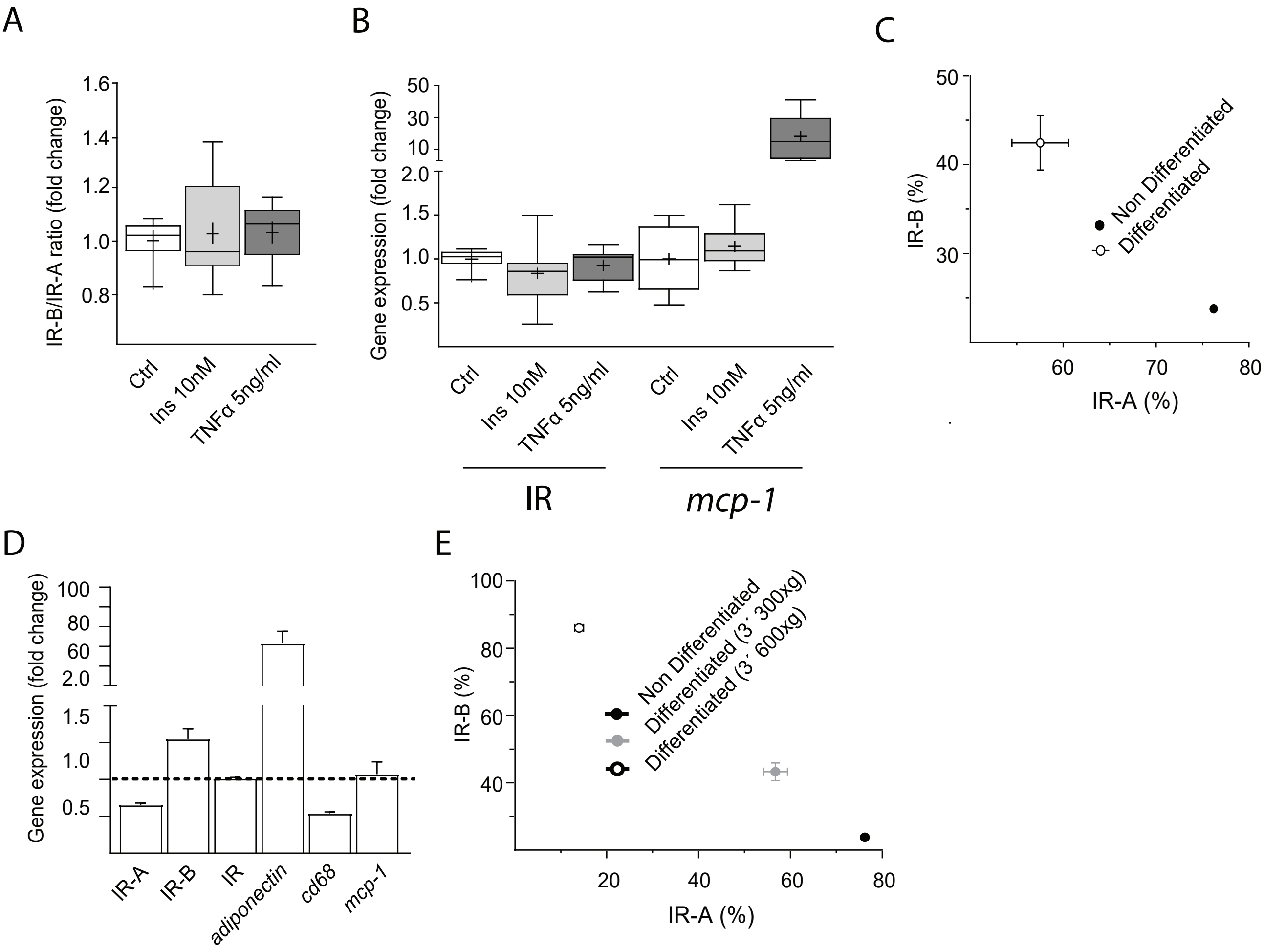

Differentiated 3T3-L1 MBX cells do not change IR isoform or total IR mRNA expression after exposure to high levels of insulin or TNFα.
3T3-L1 MBX cells were cultured in DMEM containing 4.5 g/l glucose, 2mM L-glutamine, 1mM pyruvate and supplemented with 10% heat inactivated FBS (Gibco, Carlsbad, CA, USA), penicillin 100UI/ml and streptomycin 100µg/ml (called growth media GM). Cells between passages 4-9 were incubated at 37°C, 5% CO_2, 95% humidity. For differentiation, the cells were grown until confluence and left for a further 24h, before stimulating the cells with differentiation medium I composed of GM plus 1µg/ml bovine insulin, 0.5mM IBMX, 0.25µM dexamethasone and 2µM rosiglitazone. After 48h the medium was replaced with differentiation media II composed of GM plus 1µg/ml bovine insulin for another 48h before the start of experiments. Experimental treatments consisted of GM alone or supplemented with 10nM bovine insulin or 5ng/ml TNFα for 48h. After this time, the cells were detached, centrifuged at 500xg 5min and the f loating/semi-floating fraction was combined with TRIZOL for mRNA extraction.
(A) IR-B/IR-A ratio in differentiated 3T3-L1 MBX cells without (black empty box) or with treatment with 10nM bovine insulin (light grey box) or 5ng/ml TNFα (dark grey box) for 48h as described above. Data are presented as mean, median and 10-90 percentiles of n≥6 of 3 experiments. **(B)** IR and *mcp-1* gene expression in differentiated 3T3-L1 MBX cells without (black empty box) or with treatment with 10nM bovine insulin (light grey box) or 5ng/ml TNFα (dark grey box) for 48h as described above. Data are presented as mean, median and 10-90 percentiles of n≥6 of 3 experiments. **(C)** IR-B/IR-A ratio in differentiated (white) versus non differentiated (black) from all 3T3-L1 MBX cells when TRIZOL was added directly in the well. Data are presented as mean, sem of n=2. **(D)** Gene expression changes in differentiated versus non differentiated (dotted line) 3T3-L1 MBX cells as in (C) (n=2). Data are presented as mean, sem of n=2. **(E)** IR-B/IR-A ratio in differentiated (white, grey) after floating cells collected after different centrifugation times and force to enrich for the differentiated lipid-rich cells versus non differentiated (black) from 3T3-L1 MBX cells. Data are presented as mean, sem of n=2.

Figure S5

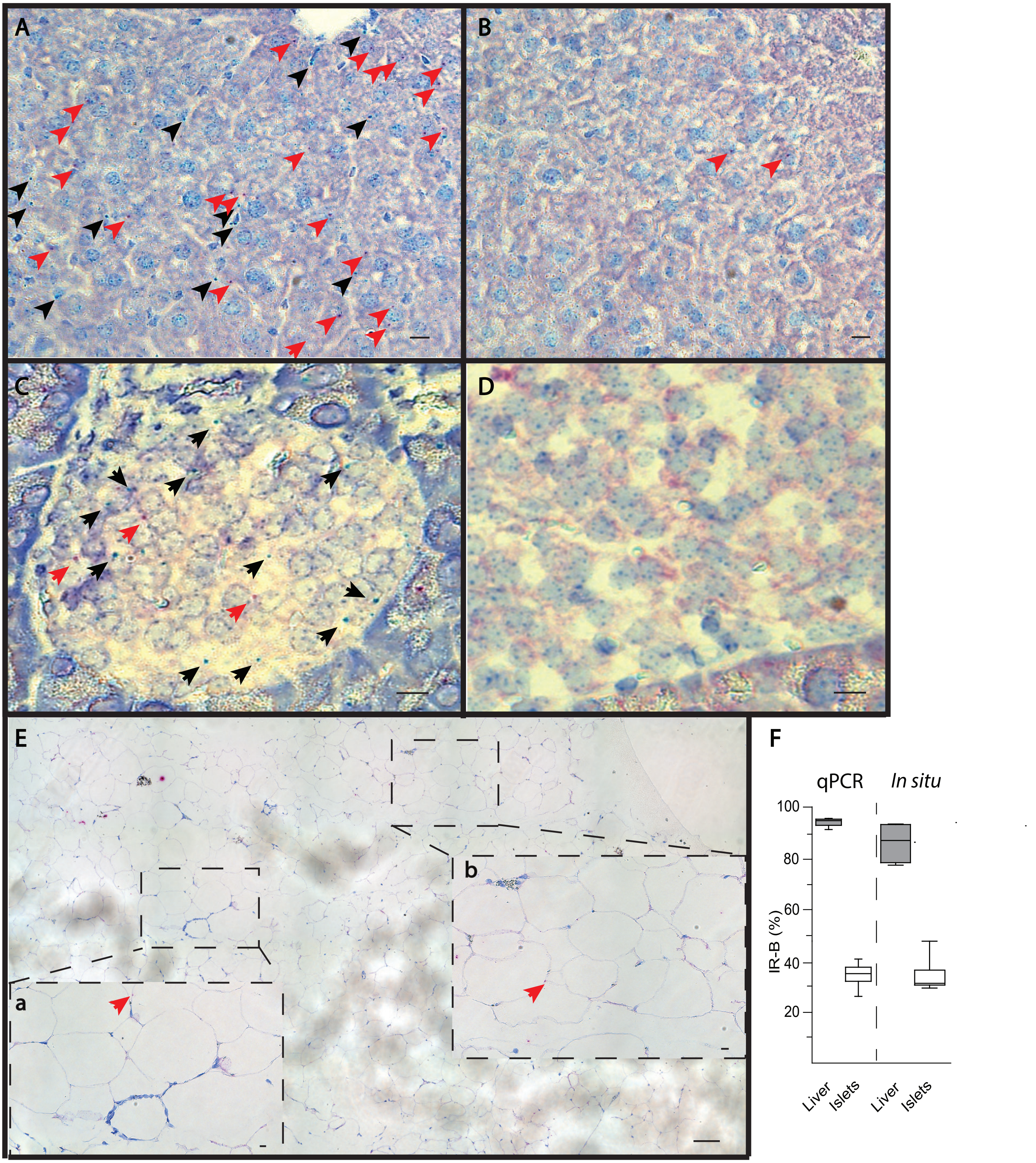

Visualizing the IR isoform mRNA *in situ* at cellular resolution.
(A) Representative *in situ* hybridization image of a liver section of a control diet mouse from the HFHSD study using IR-A and IR-B specific probes. Arrowheads indicate single IR mRNA molecules (dots): black = IR-A; red = IR-B. **(B)** Representative *in situ* hybridization image of a liver section of a control diet mouse from the HFHSD study using bacterial *dapb* probe as a negative control. Arrowheads indicate possible false positives dots of IR-B mRNA. **(C)** Representative *in situ* hybridization image of a pancreatic islet section of a control diet mouse from the HFHSD study using IR-A and IR-B specific probes. Arrowheads indicate single IR mRNA molecules (dots): black = IR-A; red = IR-B. **(D)** Representative *in situ* hybridization image of a pancreatic islet section of a control diet mouse from the HFHSD study using bacterial *dapb* probe as a negative control. No false positive dots were detected. **(E)** Representative *in situ* hybridization image of a pGAT section of a HFHSD mouse using bacterial *dapb* probe as a negative control. (a-b) Arrowheads in blow-ups indicate possible false positive dots detected throughout the whole image. **(F)** Comparison of IR-B quantification results obtained with qPCR (Figure 2 HFHSD study) and *in situ* hybridization in liver and pancreatic islet samples of control mice from the HFHSD study (n≥2). The black empty boxes represent the median and 10-90 percentiles of IR-B percentage in the measured sections (n≥4). Every single n represents a single section and is the average from at least two pictures. (A-D) Scale bar: 10µm, (E) 100µm.

Table S1 Primers list

Gene	*Sequence*
Act	F_CTAAGGCCAACCGTGAAAAG<br>R_ACCAGAGGCATACAGGGACA
B2m	F_ATGCTGAAGAACGGGAAAAA<br>R_CAGTCTCAGTGGGGGTGAAT
Cd3	F_AAGTAATGAGCTGGCTGCGT<br>R_ATGTTCTCGGCATCGTCCTG
Cd68	F_ CCACAGTTTCTCCCACCACA<br>R_GTGTAGTTCCCAAGAGCCCC
F4/80	F_ TTTCCTCGCCTGCTTCTTC<br>R_ CCCCGTCTCTGTATTCAACC
Hmbs	F_CGGAGTCATGTCCGGTAAC<br>R_GGTGCCCACTCGAATCAC
IR-A	F_ TTTTTGTCCCCAGGCCAT<br>R_CCTGTGCTCCTCCTGACTTG
IR-B	F_AACCTCTTCAGGCAATGGTG<br>R_AGGAGACGTTGGGGAAATCT
IR	F_CCTTCGAGGATTACCTGCAC<br>R_GTGTGGTGGCTGTCACATTC
IR used to generate amplicons	F_CCCCAGGCCATCCCGAA<br>R_TGTGCTCCTCCTGACTTGT
Ppia	F_GGGTTCCTCCTTTCACAGAA<br>R_GATGCCAGGACCTGTATGCT
Mcp-1	F_CTTCTGGGCCTGCTGTTC<br>R_GAGTAGCAGCAGGTGAGTGGG
Tbp	F_TGCTGTTGGTGATTGTTGGT<br>R_CTGGCTTGTGTGGGAAAGAT

Table S2 Assessment of reference genes across tissues in obesity/T2DM.

Tissue	Gene	Fold change + CI	Number n	Standard deviation (all groups)	p value	Organ	Gene	Fold change + CI	Number n	Standard deviation (all groups)	p value
**pGAT**	***hmbs***	/	22	/		**Islets**	***hmbs***	/	18	/	
	tbp	1.13±0.12	22	0.223	**n.s.**		***tbp***	0.95±0.10	18	0.150	**n.s.**
	act	1.92±0.31	6	0.543	***		*act*	1.45±0.70	6	0.525	**n.s.**
	b2m	1.77±0.24	8	0.452	***		*b2m*	1.15±0.32	8	0.348	**n.s.**
	ppia	1.72±0.46	7	0.578	***		*ppia*	0.93±0.19	4	0.107	**n.s**
**BAT**	***hmbs***	/	19	/		**Hypothalamus**	***hmbs***	/	14	/	
	tbp	1.00±0.07	19	0.178	**n.s.**		***tbp***	1.02±0.17	14	0.242	**n.s.**
	act	1.82±0.42	6	0.512	***		*act*	n.t.			
	b2m	1.81±0.28	7	0.846	***		*b2m*	n.t.			
	ppia	1.06±0.22	7	0.171	**n.s.**		*ppia*	0.98±0.20	12	0.260	**n.s.**
**SAT**	***hmbs***	/	19	/		**Heart**	***hmbs***	/	16	/	
	tbp	0.86±0.13	19	0.253	**n.s.**		***tbp***	0.90±0.10	16	0.203	**n.s.**
	act	2.59±1.30	3	0.957	***		*act*	0.79±0.08	8	0.210	**n.s.**
	b2m	1.46±0.31	12	0.448	**		*b2m*	0.88±0.45	8	0.415	**n.s.**
	ppia	1.01±0.13	4	0.054	**n.s.**		*ppia*	n.t.			
**Liver**	***hmbs***	/	24	/		**Gastrocnemius**	***hmbs***	/	15	/	
	tbp	0.69±0.08	10	0.200	*		***tbp***	0.97±0.07	15	0.128	**n.s.**
	act	1.02±0.10	24	0.205	**n.s.**		*act*	0.95±0.20	3	0.127	**n.s.**
	b2m	1.15±0.15	18	0.250	**n.s.**		*b2m*	1.28±0.25	8	0.267	**
	ppia	n.t.					*ppia*	0.90±0.24	4	0.199	**n.s.**
**Soleus**	***hmbs***	/	15	/		**Kidney**	***hmbs***	/	22	/	
	tbp	1.17±0.18	12	0.364	**n.s.**		***tbp***	1.16±0.17	21	0.316	**n.s.**
	act	1.80±0.40	8	0.655	***		*act*	0.90±0.22	6	0.225	**n.s.**
	b2m	1.31±0.71	4	0.353	**n.s.**		*b2m*	1.34±0.22	12	0.352	*
	ppia	n.t.					*ppia*	0.87±0.17	4	0.121	**n.s.**

pGAT = perigonadal adipose tissue; BAT = brown adipose tissue; SAT = subcutaneous adipose tissue. *Hmbs* = hydroxymethylbilane synthase; *tbp* = TATA-binding protein; *act* = beta actin; *b2m* = beta-2-microglobulin; *ppia* = peptidylprolyl isomerase A

Numerical presentation of fold change and confidence intervals between potential reference genes and *hmbs* gene in dietary mouse models (HFD and HFHSD) and their controls. *n* represents the number of samples in each group. The standard deviation is calculated by grouping the samples from dietary mouse models (HFD and HFHSD) and comparing them to their controls to assess which is the best reference gene with the lowest scatter throughout the tissue. Significance was calculated on the fold change using One-way Anova Bonferroni post-hoc tests. * $p<0.05$, ** $p<0.01$, *** $p<0.001$,

n.s= not significant, n.t.= not tested. In **bold** are the genes used as reference for normalization of IR in Figure 2 and Figure S3.

Supplementary methods

Animal models and diets

1) C57BL/6J male mice (Charles River Laboratories Wilmington, DE, USA) fed a high fat high sucrose diet (HFHSD: 60% kcal from fat,TD.06414; Envigo, Huntingdon, UK. and 32% sucrose in tap water) and control littermates receiving a control diet (5P76; LabDiet, St.Louis, MO, USA) for 8 weeks starting at week 12; 2) C57BL/6J mice fed a high fat diet (HFD: 60% kcal from fat, Research Diets) and control littermates received a chow diet (R70; Lantmännen, Stockholm, Sweden) for 14 weeks starting at week 8; 3) 8-weeks old male BKS.Cg-Dock7m+/+Leprdb/J (*db/db*) mice and wild-type (*wt/wt*) or heterozygous (*wt/db*) littermates (Charles River) fed a chow diet; 4) 12-weeks old B6.Cg-Lepob/J (*ob/ob*) and lean controls (*ob/wt* or *wt/wt*) (Charles River) fed a chow diet (R70; Lantmännen).

WB buffer and antibodies

Tissue lysis buffer: (30mM HEPES, 5mM EDTA, 20% Glycerol, 0.1% TRITON X-100, 1mM Na_3VO_4, 0.01M NaF and 1mM $Na_4P_2O_7$ for adipose tissue and 0.42M NaCl, 1mM NaP_2O_7, 1mM DTT, 0.02M HEPES, 0.02mM NaF, 1mM Na_3VO_4, 1mM EDTA, 1mM EGTA, 2mM PMSF and 20% Glycerol, pH 7.4) supplemented with 10µg/ml of HALT protease/phosphatase inhibitor (ThermoFisher) was added. For cells, mPER lysis buffer (Thermofisher) supplemented with 10µg/ml of HALT protease/phosphatase inhibitor was used. Loading buffer NuPAGE (Thermofisher) with addition of reducing agent (DTT). Fluorescent secondary antibodies were used at 1:10000 (Li-Cor) or HRP-linked anti-Rabbit or anti-Mouse at 1:1000 (Cell signaling Danvers, MA, USA). Primary antibodies: Anti-γ-Tubulin 1:5000 (Sigma), Anti-α-Tubulin 1:5000 (Sigma), Anti-IR 1:1000 (Cell Signalling), Anti-HMBS 1:1000 (Thermofisher).

Calculations of IR percentages

The percentages of IR-A and IR-B were calculated in the following ways: 1. IR-B or IR-A mRNA expressed in percentage and normalized to total IR mRNA, by calculating the Δct between the IR-B (or IR-A) and the IR and applying the following calculation: 2-Δct *100. The IR-A (or IR-B) is then 100% minus the IR-B% (or IR-A). 2. IR-B normalized to IR-A, by calculating the Δct between IR-B and IR-A and applying the following calculation: 2-Δct (called here x). IR-B or x is expressed now as fold increase or decrease compared to IR-A which is considered 1. The total IR (100%) is x+1 (IR-B+IR-A) and the percentage of IR-B was then calculated by x*100/x+1 (IR-B*100/IR-B+IR-A). The IR-A is then 100% minus the IR-B%. The data in the Figures 1-3 and linked to results in the text were calculated

using the average of these three calculations (IR-A vs IR, IR-B vs IR and IR-A vs IR-B) apart from hypothalamus were the percentage calculated using IR-A vs IR was not used due to the close proximity of the IR-A to 100%.
