# Supplementary material for: Tissue‐specific expression of insulin receptor isoforms in obesity/type 2 diabetes mouse models
Source: J Cell Mol Med. 2021 Mar 19;25(10):4800–13. doi: 10.1111/jcmm.16452 (PMC8107091; doi:10.1111/jcmm.16452)
Supplement: Supplementary file 2 — Figure S1 [file JCMM-25-4800-s002.pdf]

Figure S1

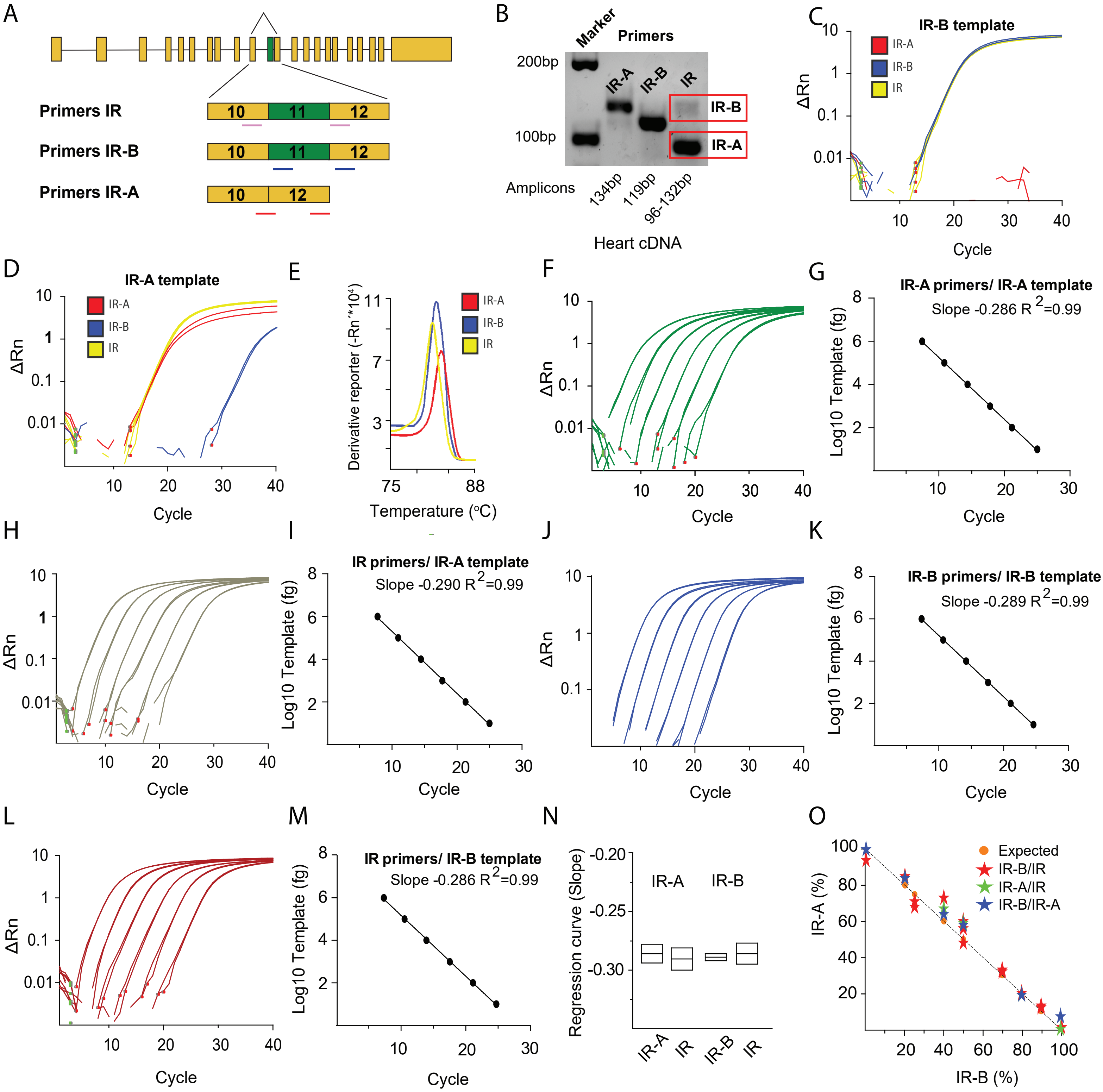

Primer specificity and selectivity for detection of total and isoform specific IR gene expression using real time qPCR.

Primer pairs for IR mRNA, amplifying both IR-A and IR-B, were designed to bind upstream and downstream of exon 11; for IR-B mRNA the forward primer binds to exon 11; for IR-A mRNA, the forward primer binds to the exon 10-12 junction **(A)**. The primers´ specificity was confirmed using mouse tissue cDNA by agarose gel electrophoresis **(B)** and melting curve analysis **(E)**. IR-B primers did not amplify IR-A template, while IR-A primers weakly amplified the IR-B template with a delay of 16 cycles **(C-D)**. Regression curves and slope calculation for each primer/template pair was carried out using the plasmids expressing IR-A and IR-B amplicons serially diluted from 1ng to 10fg **(F-M)**. To compare and standardize the calculations of IR isoforms using the ct value, an equal threshold for all three curves was chosen. The efficiency calculated for the IR-A plasmid (mean±CI for IR-A primers 93.5±2.5%; IR primers 95±4%) and the IR-B plasmid (mean±CI for IR-B primers 94.5±1.5%; IR primers 93±4%) as well as the regression slopes for each primer/template pair **(N)** did not differ among primers. We prepared different mixtures of IR-A and IR-B plasmids, containing between 0 and 100% of them respectively **(O)**. Using specific primers for the IR isoforms as well as for total IR, we measured the percentage of IR isoform amplicons amplified from the plasmid mixtures using the ct values of IR, IR-A and/or IR-B and compared the results to the predicted values (percentage of molecules used in the dilutions). The results were comparable to the expected values derived from the plasmid mixtures.

(A) Scheme of IR gene, exons (yellow and green) and the location of primers designed to recognize IR (both IR-A and IR-B; pink lines), IR-B (blue lines) and IR-A (red lines). Exon 11, which is present in IR-B and absent in IR-A, is shown in green. **(B)** Image of amplicons from real-time qPCR, performed in heart tissue of control mice, separated on a 3% agarose gel. IR primers amplify both IR isoforms (product size IR-B 132bp; IR-A 96bp; highlighted in the red rectangles). IR-A specific primers amplify the IR-A amplicon (134bp) and IR-B specific primers amplify the IR-B amplicon (119bp). **(C,D)** Real-time qPCR amplification curves using template plasmids containing the cDNA of amplicons generated using either IR-B or IR-A primers. Primers specific for IR-A (red), IR-B (blue) and IR (yellow) were used for amplification. **(E)** Melting temperatures of qPCR-products amplified from mouse tissue using primers for IR-A (red), IR-B (blue) and IR (yellow). **(F-M)** Titration of plasmids templates containing IR-A (F-I) or IR-B (J-M) amplicons using real-time qPCR and IR-A (F,G), IR-B (J,K) or IR (H,I,L,M) specific primers. Template concentration ranged from 10fg to 1ng. The regression curves and slopes were calculated by fitting the ct values of the average of duplicate measurements for IR-A primers on IR-A amplicon-containing plasmid template (G), IR-B primers on IR-B amplicon-containing plasmid template (K) and IR primers on IR-A (I) and IR-B (M) amplicon-containing plasmid templates. **(N)** Representation of mean slope and confidence interval of regression curves (n=2) generated using IR-A and IR-B amplicon-containing plasmid templates and IR primers. **(O)** Percentage of IR isoform amplicons (colored stars) obtained from a known mixture of IR-A and IR-B amplicon-containing plasmid templates (orange circles). The percentage was calculated using IR-B and IR ct values (red), IR-A and IR (green) or IR-B and IR-A (blue) as described in the method section.
The dotted line represents the expected ratio.
