# Supplementary material for: Tissue‐specific expression of insulin receptor isoforms in obesity/type 2 diabetes mouse models
Source: J Cell Mol Med. 2021 Mar 19;25(10):4800–13. doi: 10.1111/jcmm.16452 (PMC8107091; doi:10.1111/jcmm.16452)
Supplement: Supplementary file 3 — Figure S2 [file JCMM-25-4800-s006.pdf]

Figure S2

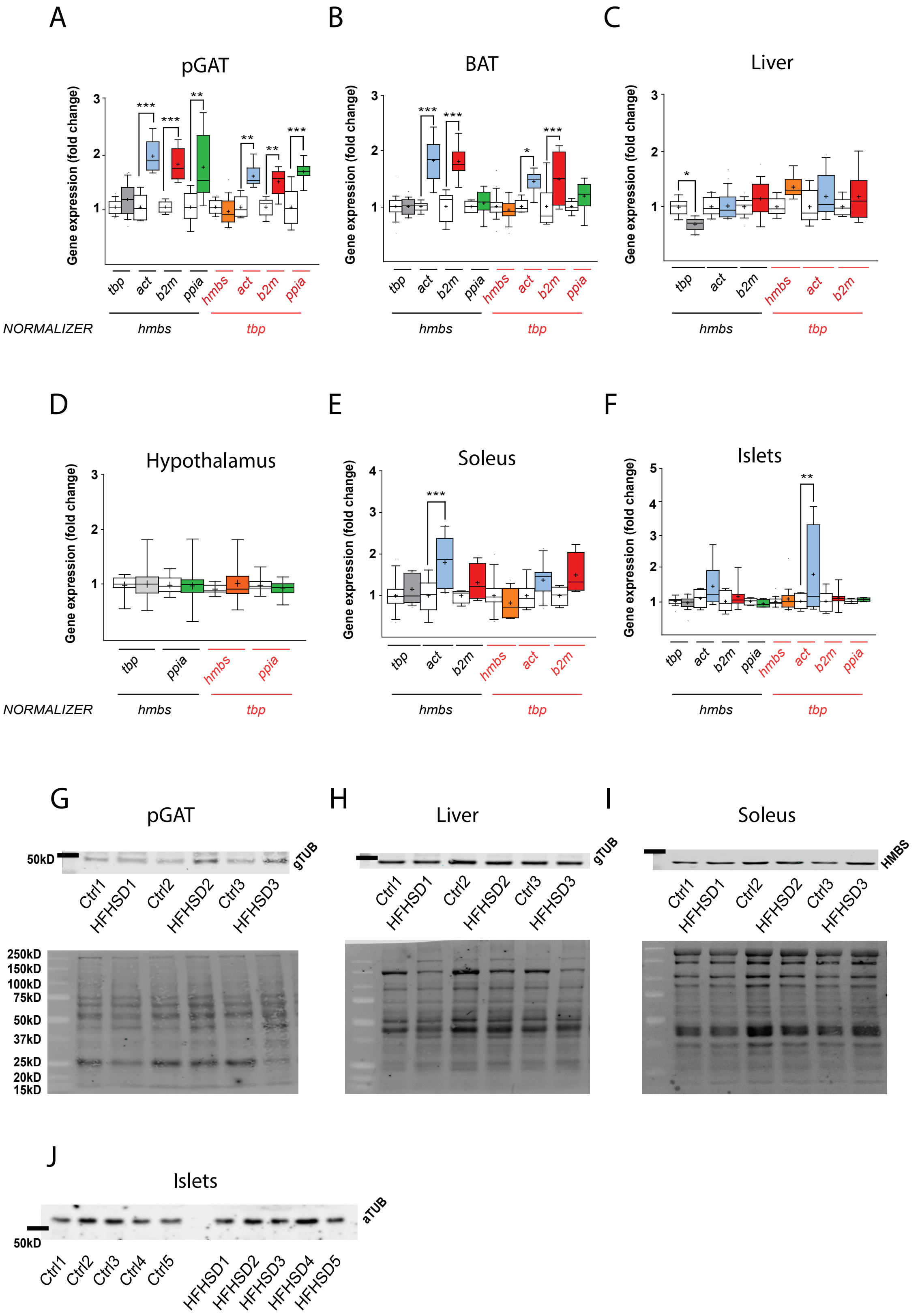

Assessment of tissue-specific reference genes and Western blot membrane protein/ normalizer for Figure 2.
(A-F) Quantification of real-time qPCR data of candidate reference genes normalized to either *hmbs* (black) or *tbp* (red) in control diet mice compared to dietary treatments (HFHSD and HFD combined). Data are presented as fold change mean, median and 10-90 percentile compared to control cohorts (black empty boxes). Boxes: grey = *tbp*; blue = *actin*; red = *b2m*; green = *ppia*; orange = *hmbs*. Statistical significance was calculated using one-way ANOVA and Bonferroni´s post test. Minimum n for each tissue: (A) perigonadal adipose tissue (pGAT) n=6; (B) brown adipose tissue (BAT) n=6; (C) liver n=9; (D) hypothalamus n=6; (E) soleus n=4; (F) isolated pancreatic islets (islets) n=7, * p<0.05 ** p<0.01 *** p<0.001.
The chosen reference genes for each tissue are bolded in Table S1.
(G-J) Representative blot of tissue selected normalizer and protein transferred used to normalize the IR in Figure 2. pGAT (G), liver (H), soleus (I) and pancreatic islets (J).
