# Supplementary material for: Tissue‐specific expression of insulin receptor isoforms in obesity/type 2 diabetes mouse models
Source: J Cell Mol Med. 2021 Mar 19;25(10):4800–13. doi: 10.1111/jcmm.16452 (PMC8107091; doi:10.1111/jcmm.16452)
Supplement: Supplementary file 4 — Figure S3 [file JCMM-25-4800-s003.pdf]

Figure S3

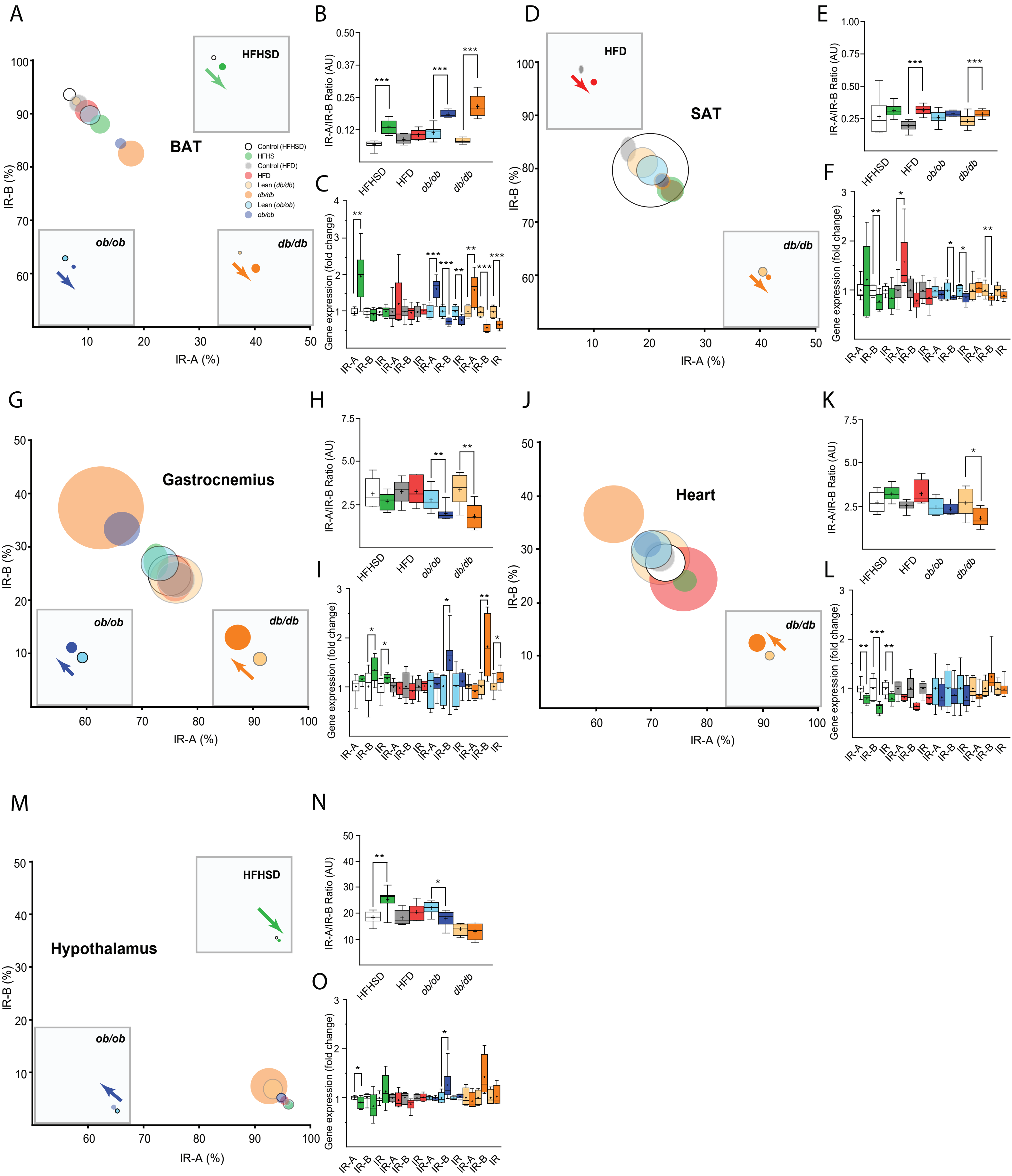

Tissue-specific changes of IR mRNA and its isoforms in obesity/T2DM

(A,D,G,J,M) Percentages of IR isoform mRNAs in brown adipose tissue (BAT) (A), subcutaneous adipose tissue (SAT) (D), gastrocnemius (G), heart (J), and hypothalamus (M) calculated using ct values from real-time qPCR and presented as confidence intervals. Insets show cohorts with significant differences, arrows indicate the direction of change. **(B,E,H,K,N)** IR-A/IR-B ratio in BAT (B), SAT (E), gastrocnemius (H), heart (K), and hypothalamus (N), calculated using ct values from real-time qPCR and presented as mean, median and 10-90 percentiles. **(C,F,I,L,O)** IR gene expression in BAT (C), SAT (F), gastrocnemius (I), heart (L), and hypothalamus (O), normalized to tissue-selected reference genes (Figure S2) and presented as fold change with mean, median and 10-90 percentiles in comparison to the control for the specific cohort.

Circles and boxes: black empty = control diet to HFHS; green = HFHSD for 8 weeks; gray = control diet to HFD; red = HFD for 14 weeks; light blue = control to *ob/ob*; blue = *ob/ob* mice 3 months old; light orange = control to *db/db*; orange = *db/db* mice 8 weeks old. * p<0.05 ** p<0.01 *** p<0.001. BAT n≥5; SAT n=8; Gastrocnemius n≥7; Heart n≥4, Hypothalamus n≥4.
