# Supplementary material for: Tissue‐specific expression of insulin receptor isoforms in obesity/type 2 diabetes mouse models
Source: J Cell Mol Med. 2021 Mar 19;25(10):4800–13. doi: 10.1111/jcmm.16452 (PMC8107091; doi:10.1111/jcmm.16452)
Supplement: Supplementary file 5 — Figure S4 [file JCMM-25-4800-s005.pdf]

Figure S4

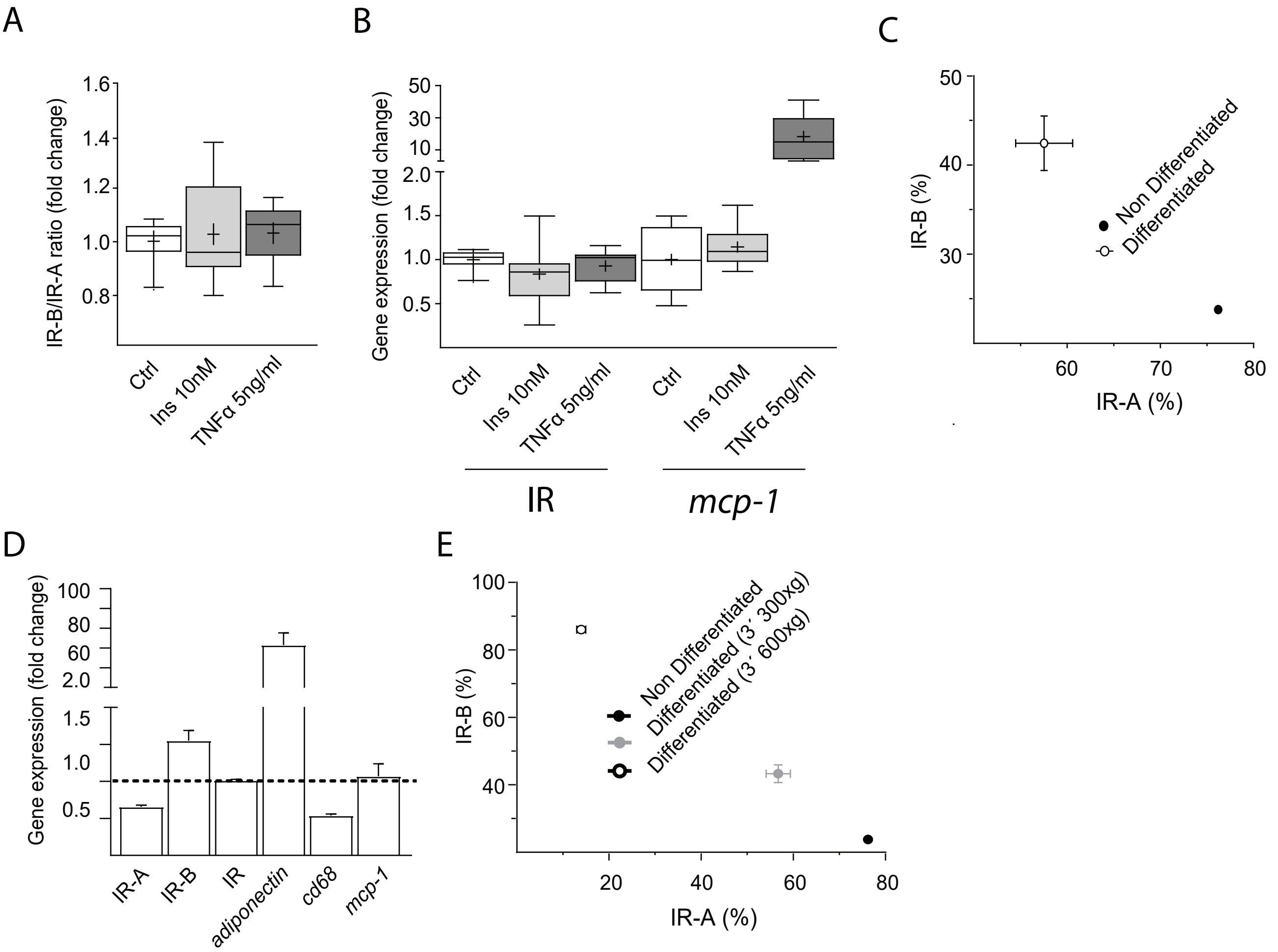

Differentiated 3T3-L1 MBX cells do not change IR isoform or total IR mRNA expression after exposure to high levels of insulin or TNFα.
3T3-L1 MBX cells were cultured in DMEM containing 4.5 g/l glucose, 2mM L-glutamine, 1mM pyruvate and supplemented with 10% heat inactivated FBS (Gibco, Carlsbad, CA, USA), penicillin 100UI/ml and streptomycin 100µg/ml (called growth media GM). Cells between passages 4-9 were incubated at 37°C, 5% CO_2, 95% humidity. For differentiation, the cells were grown until confluence and left for a further 24h, before stimulating the cells with differentiation medium I composed of GM plus 1µg/ml bovine insulin, 0.5mM IBMX, 0.25µM dexamethasone and 2µM rosiglitazone. After 48h the medium was replaced with differentiation media II composed of GM plus 1µg/ml bovine insulin for another 48h before the start of experiments. Experimental treatments consisted of GM alone or supplemented with 10nM bovine insulin or 5ng/ml TNFα for 48h. After this time, the cells were detached, centrifuged at 500xg 5min and the f loating/semi-floating fraction was combined with TRIZOL for mRNA extraction.
(A) IR-B/IR-A ratio in differentiated 3T3-L1 MBX cells without (black empty box) or with treatment with 10nM bovine insulin (light grey box) or 5ng/ml TNFα (dark grey box) for 48h as described in the method section. Data are presented as mean, median and 10-90 percentiles of n≥6 of 3 experiments. **(B)** IR and *mcp-1* gene expression in differentiated 3T3-L1 MBX cells without (black empty box) or with treatment with 10nM bovine insulin (light grey box) or 5ng/ml TNFα (dark grey box) for 48h as described in the method section. Data are presented as mean, median and 10-90 percentiles of n≥6 of 3 experiments. **(C)** IR-B/IR-A ratio in differentiated (white) versus non differentiated (black) from all 3T3-L1 MBX cells when TRIZOL was added directly in the well. Data are presented as mean, sem of n=2. **(D)** Gene expression changes in differentiated versus non differentiated (dotted line) 3T3-L1 MBX cells as in (C) (n=2). Data are presented as mean, sem of n=2. (E) IR-B/IR-A ratio in differentiated (white, grey) after floating cells collected after different centrifugation times and force to enrich for the differentiated lipid-rich cells versus non differentiated (black) from 3T3-L1 MBX cells. Data are presented as mean, sem of n=2.
