# Supplementary material for: Tissue‐specific expression of insulin receptor isoforms in obesity/type 2 diabetes mouse models
Source: J Cell Mol Med. 2021 Mar 19;25(10):4800–13. doi: 10.1111/jcmm.16452 (PMC8107091; doi:10.1111/jcmm.16452)
Supplement: Supplementary file 6 — Figure S5 [file JCMM-25-4800-s001.pdf]

Figure S5

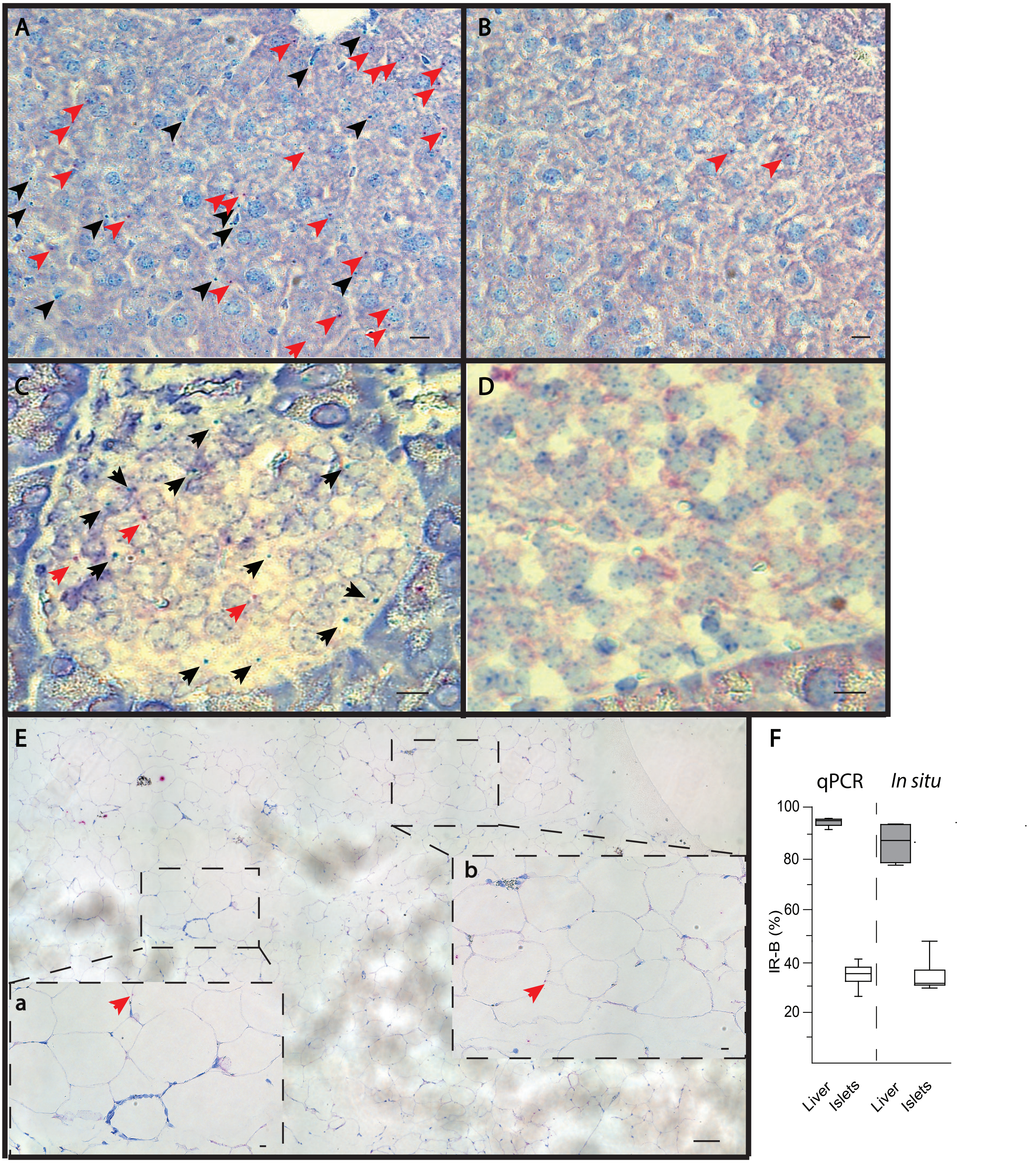

Visualizing the IR isoform mRNA *in situ* at cellular resolution.
(A) Representative *in situ* hybridization image of a liver section of a control diet mouse from the HFHSD study using IR-A and IR-B specific probes. Arrowheads indicate single IR mRNA molecules (dots): black = IR-A; red = IR-B. **(B)** Representative *in situ* hybridization image of a liver section of a control diet mouse from the HFHSD study using bacterial *dapb* probe as a negative control. Arrowheads indicate possible false positives dots of IR-B mRNA. **(C)** Representative *in situ* hybridization image of a pancreatic islet section of a control diet mouse from the HFHSD study using IR-A and IR-B specific probes. Arrowheads indicate single IR mRNA molecules (dots): black = IR-A; red = IR-B. **(D)** Representative *in situ* hybridization image of a pancreatic islet section of a control diet mouse from the HFHSD study using bacterial *dapb* probe as a negative control. No false positive dots were detected. **(E)** Representative *in situ* hybridization image of a pGAT section of a HFHSD mouse using bacterial *dapb* probe as a negative control. (a-b) Arrowheads in blow-ups indicate possible false positive dots detected throughout the whole image. **(F)** Comparison of IR-B quantification results obtained with qPCR (Figure 2 HFHSD study) and *in situ* hybridization in liver and pancreatic islet samples of control mice from the HFHSD study (n≥2). The black empty boxes represent the median and 10-90 percentiles of IR-B percentage in the measured sections (n≥4). Every single n represents a single section and is the average from at least two pictures. (A-D) Scale bar: 10µm, (E) 100µm.
